# Stereoselective Copper-Catalyzed Cross-Coupling of α-CF_3_-Allylboronic Acids with Diazoketones

**DOI:** 10.1021/acs.joc.4c02869

**Published:** 2025-01-30

**Authors:** Tautvydas Kireilis, Kálmán J. Szabó

**Affiliations:** Department of Organic Chemistry, Stockholm University, SE-106 91 Stockholm, Sweden

## Abstract

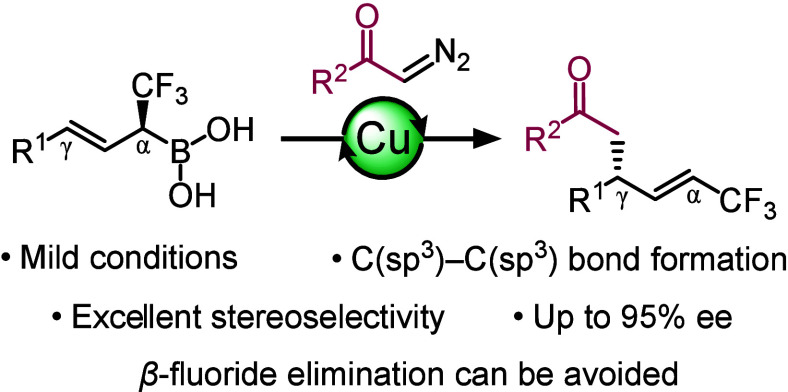

We have studied copper-catalyzed cross-coupling of chiral α-CF_3_-substituted allylboronic acids with α-diazoketones. The reaction proceeds with excellent regioselectivity and stereoselectivity via allylic rearrangement. The method is useful for formation a new allylic C(sp^3^)–C(sp^3^) bond with high selectivity. The poor yield is a limitation of this reaction.

Allylboronates are very attractive reagents in selective organic synthesis.^1,2^ Allylboration of carbonyl compounds,^3^ indoles, imines and their congeners^4−11^ is an efficient approach for synthesis of stereodefined homoallylic alcohols and amines.

Another emerging area is selective cross-coupling reactions involving allylboronates. A particularly interesting feature of these processes is formation of C(sp^3^)–C(sp^2^) and even C(sp^3^)–C(sp^3^) bonds, which is usually challenging in transition metal catalysis because of β-hydride elimination related issues. Suzuki–Miyaura-type coupling of allylboronates and aryl halides received particularly broad attention^12−16^ because of the possibility of control of regio- and stereochemistry of the reaction (e.g., Scheme 1a). Sawamura and co-workers^17^ published a study on copper-catalyzed asymmetric allyl–allyl coupling involving allyl-Bpin species (Scheme 1b). Stereoselective transition metal catalyzed transformation of chiral α-CF_3_ boronates is particularly challenging. The reason is that transmetalation of the boronates leads to formation of a carbon–metal bond, which rapidly undergoes β-fluoride elimination removing the stereocenter (Scheme 2a).^18^ We reasoned that the degradation of the stereocenter can be avoided by transition metal catalyzed cross-coupling via allylic rearrangement (Scheme 2b). We have previously studied the cross-coupling reactions of diazoketones and allylboronic acids, which is suitable for formation of C(sp^3^)–C(sp^3^) bonds.^19,20^ The copper catalyzed version of this process occurs stereoselectively with an allylic rearrangement, which was demonstrated using racemic allylboron species. In this note, we present our results using diazoketones and chiral α-CF_3_ allylboronic acids as substrates for this copper-catalyzed cross-coupling reaction (Scheme 1c).

**Scheme 1:**
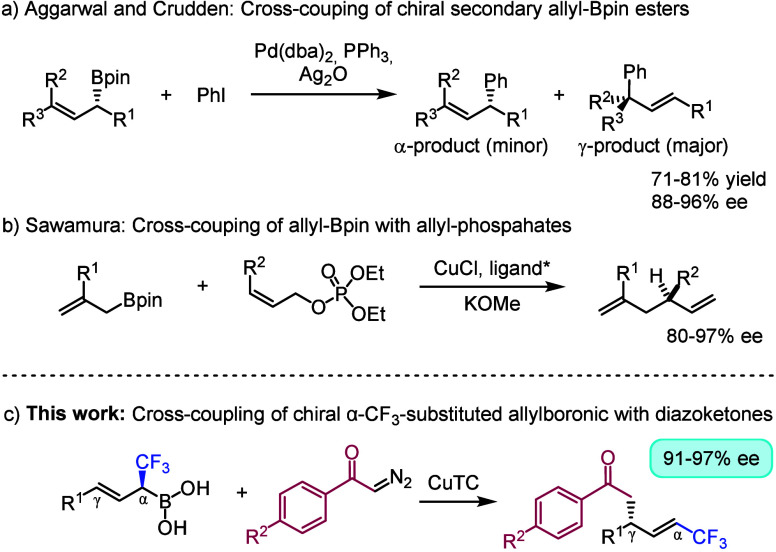
Examples for Metal-Catalyzed Cross-Coupling Involving Allylboronates

**Scheme 2:**
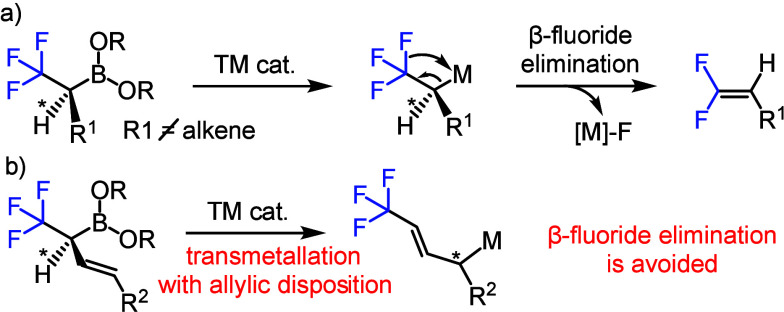
Concept for Stereoselective Transition Metal Catalyzed Transformation of α-CF_3_ Boronates

We have found (Table 1, entry 1) that the CuTC catalyzed cross-coupling of chirally enriched allylboronic acid **1a** and diazoketone **2a** gives alkenyl-CF_3_ compound **4a** involving a C(sp^3^)–C(sp^3^) bond with high selectivity (95% ee). Analysis of the NMR spectra indicated the selective formation of the Z-isomer of the alkenyl-CF_3_ group. Although, the selectivity was high, the yield was poor (38%). We have found that the starting material **1a** was consumed (<5% remained) but a complex reaction mixture was formed with unidentifiable, often volatile byproducts with similar (low) polarity. Interestingly, defluorination compounds with C=CF_2_ groups were not found by analysis of the ^1^H- and ^19^F-NMR spectra. We made numerous attempts to improve the yields; however, these efforts remained fruitless. Addition of molecular sieves had no significant effect on the reaction (entry 2). Cross-coupling product **4a** did not form by using the Bpin form of **1a** (entry 3). We have also tested a couple of other copper catalysts. No reaction takes place with CuI (entry 4), while Cu(acac)_2_ is a competent catalyst (entry 5) but inferior to CuTC. Increase of the catalyst loading from 10 to 20 mol % had very small effect on the outcome of the cross-coupling reaction. Increasing the concentrations (by using less solvent) also had adverse effects on the yield (entry 8). DCM proved to be the best solvent for the reaction. When DCM was replaced with toluene, the yield and even the selectivity were decreased (entry 9). Acetonitrile was not effective (entry 10). Increase of the reaction temperature from rt to 40 °C or elongation of the reaction time did not improve the yield. Simultaneous addition of both boronic acid and diazoketone over 2 h did not increase the yield (entry 13). Formation of **4a** was not detected, when the reaction was conducted in the absence of copper catalyst (entry 14).

**Table 1:**
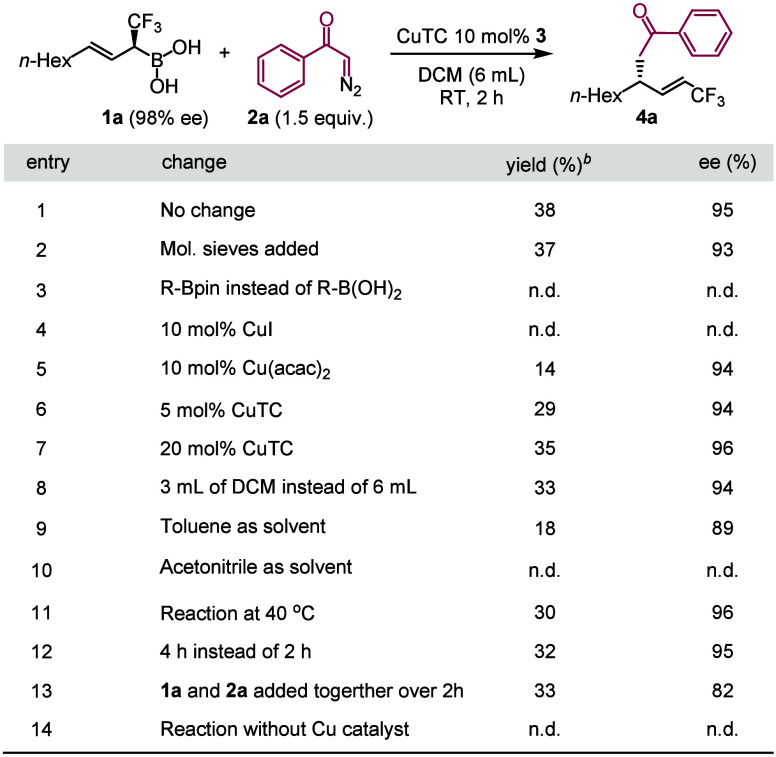
Optimal Conditions for the Cu-Catalyzed Cross-Coupling Reaction^a^

a Boronic acid **1a** (23.8 mg, 0.1 mmol) and CuTC **3** (1.9 mg, 0.01 mmol) were dissolved in 5 mL of DCM. While the mixture was stirring, diazoketone **2a** (21.9 mg, 0.15 mmol in 1 mL of DCM) was added dropwise over 1.5 h followed by another 0.5 h of stirring. All reactions were performed under inert atmosphere at RT.

b Isolated yields. n.d. = not detected.

We have also varied the diazoketone component in the reaction (Table 2, entries 1–5). The reactions proceeded with high levels of chirality transfer, giving alkenyl-CF_3_ products with a new allylic C(sp^3^)–C(sp^3^) bond. However, the yields were poor. The problem was the same, as with **2a** as the diazoketone component (entry 1), the formation of large amounts of unpolar byproducts, which could not be identified. When the reaction was repeated on 1 mmol scale the selectivity was high (93% ee) but the yield (35%) was about as poor as in smaller scale (entry 1). Variation of the aromatic substituents had a relatively small effect on the yield and selectivity. Diazoketone precursors with aromatic ^t^Bu, **2b** (entry 2), bromo, **2c** (entry 3) and CF_3_, **2e** (entry 5) substituents gave about the same yield as with **1a** (entry 1). Fluoro derivative **2d** could be coupled with a yield of 45% (entry 4). We also briefly studied the replacement of the allylic hexyl (**1a**) with a phenethyl (**1b**) moiety. The cross-coupling reaction of **1b** with **2a**–**b** proceeds still with high selectivities, but the yields were poor (entries 6–7). Other types of α-CF_3_ allylboronic acids suffered from stability and reactivity issues under the conditions of cross-coupling with diazoketones.

**Table 2:**
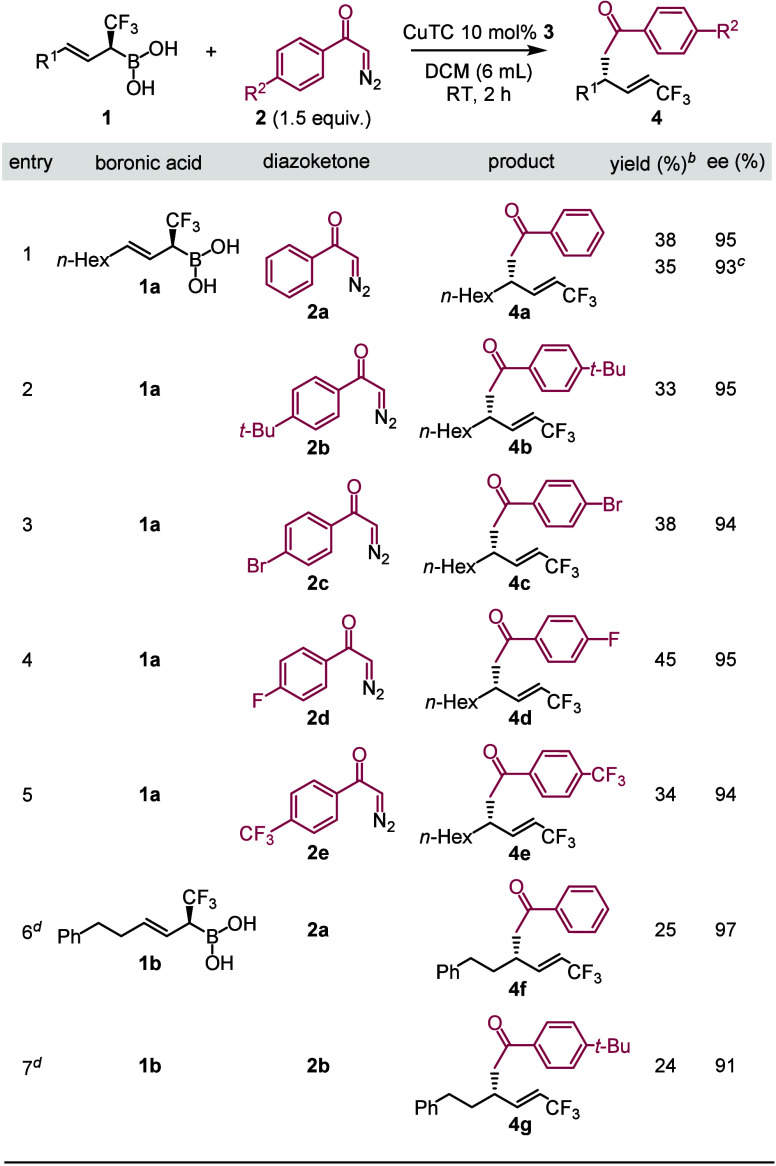
Substrate Scope of Cu-Catalyzed Coupling^a^

a Unless otherwise stated, boronic acid **1a**–**b** (0.1 mmol), CuTC (0.01 mmol, 0.1 equiv) were dissolved in DCM (5 mL), and diazoketone **2a**–**e** (0.15 mmol, 1.5 equiv in 1 mL of DCM) added over 1.5 h.

b Isolated yields.

c Reaction performed at 1 mmol scale.

d Diazoketone was added dropwise over a period of 3 h.

Based on the above results and previous studies^19,20^ we propose a mechanism for the cross-coupling reaction of diazoketones and α-CF_3_ allylboronic acids (Scheme 3). The first step of this process is supposed to be formation of copper carbene **5** from CuTC (**3**) and the diazoketone precursor (**2**).^21,22^ Subsequently, this copper carbene intermediate coordinates to α-CF_3_ allylboronic acid reagent **1** to give complex **6**. Our assumption is that this coordination is facilitated by the interaction of the B(OH)_2_ group of **1** with the copper of the carbene. The importance of a similar type of M–O(H)–B type of interaction was pointed out for transmetalation of organoboronates in Suzuki–Miyaura reactions.^23^ A possible explanation of the failure for using allyl-Bpin compound as a boronate precursor (Table 1, entry 3) may explain that (under base free conditions) such an M–O(H)–B type interaction cannot be formed. The displacement of the boron with formation of the copper–carbon bond takes place with allylic disposition. As mentioned above, allylic disposition prevents formation of CF_3_–CH–M bond, which would lead to rapid defluorination of the CF_3_ group (see Scheme 2). The above results indicate a high level of chirality transfer, and our previous studies^19,20^ indicated a syn selective S_E_2′ displacement of the boron. Assignment of the configuration in **4a**–**g** is also based on these findings.

**Scheme 3:**
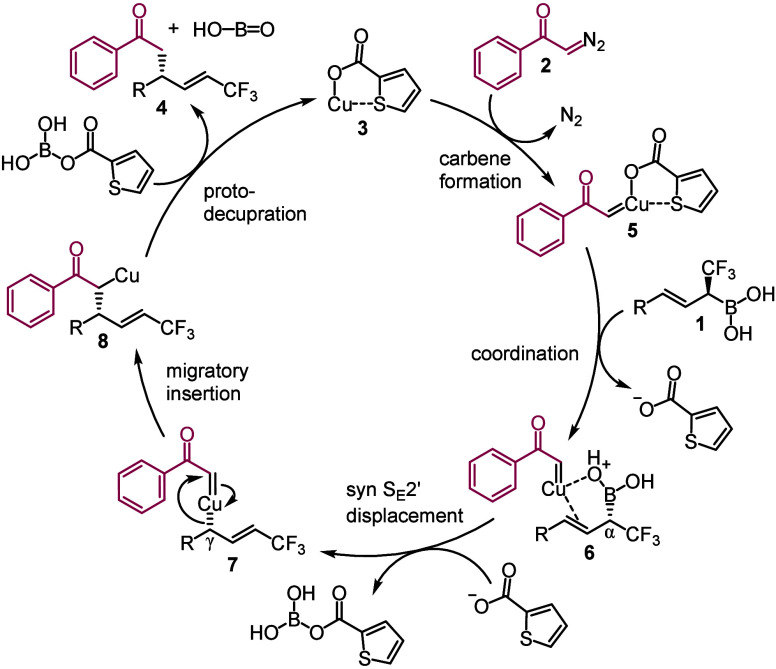
Proposed Reaction Mechanism for Cu-Catalyzed Cross-coupling of Allylboronic Acids with α-Diazoketones

The next step is probably a migratory insertion of allyl copper-carbene **7** to **8**. A similar type of insertion reaction was described by Wang and co-workers^24^ for migratory insertions of palladium-carbenes. This special type of migratory insertion contributes to the β-hydride elimination-free formation of a new C(sp^3^)–C(sp^3^) bond. The last step is supposed to be proto decupration of **8** probably via enolization of the carbonyl group. Possibly, this is the reason that the coupling reaction can be carried out only with diazoketone reagents.

In summary, we have developed a method for the stereoselective cross-coupling of chirally enriched α-CF_3_-substituted allylboronic acids with α-diazoketones. The reaction proceeds with excellent levels of chirality transfer via allylic disposition. An interesting feature is that this transition metal catalyzed cross-coupling reaction takes place without β-defluorination and dehydrogenation forming a new allylic C(sp^3^)–C(sp^3^) bond. A further interesting feature is that the reaction takes place under base-free conditions at rt with allylboronic acids, but no reaction takes place with the Bpin analogue. Although the synthetic value of the reaction is limited by the poor yields, this paper discloses a number of interesting stereochemical and reactivity features of this cross-coupling reaction.

## Experimental Section

### General Information

All reactions were performed under an inert atmosphere in oven-dried glassware. All solvents were degassed by nitrogen and dried over 4 Å molecular sieves. Allylboronic acids **1a**–**b**^5^ and diazoketones **2a**–**e**^25^ were prepared according to the published procedures. The remaining of the reagents were received from commercial sources. The isolated compounds were characterized by ^1^H, ^13^C and ^19^F NMR spectroscopy using Bruker 400 MHz spectrometers. The ^1^H NMR experiments were reported in units, parts per million (ppm), and were measured relative to the signals for residual chloroform (7.26 ppm) in the deuterated solvent. All ^13^C NMR spectra were reported in parts per million relative to deuterated chloroform (77.16 ppm). High resolution mass spectrometry (HRMS) was performed using the ESI technique and a time-of-flight detector.

### General Procedure. Stereoselective Cu-Catalyzed Coupling of α-CF_3_-Allylboronic Acids with α-Diazoketones

An oven-dried reaction flask was brought into the glovebox and loaded with CuTC catalyst **3** (0.01 mmol, 0.1 equiv), 4.75 mL of dichloromethane and allylboronic acid **1** (0.1 mmol in 0.25 mL of DCM). To this reaction mixture, diazoketone **2** (0.15 mmol, 1.5 equiv, in 1 mL of DCM) was added dropwise over 1.5 h followed by another 0.5 h of stirring. The final reaction concentration was 0.017 mol/L. Product **4** was isolated by silica gel chromatography.

#### (R,E)-1-Phenyl-3-(3,3,3-trifluoroprop-1-en-1-yl)nonan-1-one (**4a**)

This compound was prepared according to the above general procedure. Product **4a** was isolated as a colorless liquid (11.8 mg, 38%) by silica gel chromatography using a pentane/dichloromethane 6:1 solvent system as an eluent. Large scale reaction was perfomed by dissolving boronic acid **1a** (238 mg, 1 mmol), CuTC (19.1 mg, 0.1 mmol) in DCM (59 mL) and **2a** (219 mg, 1.5 mmol in 1 mL) was added over 1.5 h, followed by another 0.5h of stirring. Products **4a** was isolated as colorless liquid (109 mg, 35%) by silica gel chromatography using pentane/dichloromethane 6:1 solvent system as eluent ^1^H NMR (400 MHz, CDCl_3_) δ 7.92–7.94 (m, 2H), 7.55–7.60 (m, 1H),7.45–7.49 (m, 2H), 6.27 (ddq, *J* = 15.8, 8.6, 2.2 Hz, 1H), 5.65 (dq, *J* = 15.8, 6.3 Hz, 1H), 2.98–3.10 (m, 2H), 2.87–2.96 (m, 1H), 1.50–1.56 (m, 1H), 1.37–1.46 (m, 1H), 1.23–1.32 (m, 8H), 0.88 (t, *J* = 6.7 Hz, 3H). ^13^C{^1^H} NMR (101 MHz, CDCl_3_) δ 198.2, 143.4 (q, *J* = 6.5 Hz), 137.1, 133.4, 128.8, 128.2, 123.2 (d, *J* = 269.2 Hz), 118.9 (d, *J* = 33.3 Hz), 43.1, 37.3, 34.2, 31.8, 29.3, 27.1, 22.7, 14.2. ^19^F NMR (377 MHz, CDCl_3_) δ −63.89 (dt, *J* = 6.1, 1.4 Hz). HRMS (ESI-TOF) *m*/*z*: [M + Na]^+^ Calcd for C_18_H_23_F_3_ONa 335.1593; Found 335.1593.

#### (R,E)-1-(4-(*tert*-Butyl)phenyl)-3-(3,3,3-trifluoroprop-1-en-1-yl)nonan-1-one (**4b**)

This compound was prepared according to the above general procedure. Product **4b** was isolated as a colorless liquid (12.0 mg, 33%) by silica gel chromatography using a pentane/dichloromethane 4:1 solvent system as eluent. ^1^H NMR (400 MHz, CDCl_3_) δ 7.87 (d, *J* = 8.6 Hz, 2H), 7.48 (d, *J* = 8.6 Hz, 2H), 6.27 (ddq, *J* = 15.8, 8.4, 2.1 Hz, 1H), 5.65 (dqd, *J* = 15.8, 6.4, 1.0 Hz, 1H), 2.95–3.07 (m, 2H), 2.87–2.93 (m, 1H), 1.49–1.55 (m, 1H), 1.39–1.45 (m, 1H), 1.34 (s, 9H), 1.24–1.31 (m, 8H), 0.87 (t, *J* = 6.3 Hz, 3H). ^13^C{^1^H} NMR (101 MHz, CDCl_3_) δ 197.8, 157.1, 143.4 (q, *J* = 6.3 Hz), 118.8 (q, *J* = 33.4 Hz), 134.5, 128.1, 125.7, 123.1 (q, *J* = 268.9 Hz), 118.8 (q, *J* = 33.4 Hz), 42.9, 37.2, 35.2, 34.1, 31.8, 31.2, 29.3, 27.1, 22.7, 14.2. ^19^F NMR (377 MHz, CDCl_3_) δ −63.86 (dt, *J* = 6.6, 1.8 Hz). HRMS (ESI-TOF) *m*/*z*: [M + Na]^+^ Calcd for C_22_H_31_F_3_ONa 391.2219; Found 391.2220.

#### (R,E)-1-(4-Bromophenyl)-3-(3,3,3-trifluoroprop-1-en-1-yl)nonan-1-one (**4c**)

This compound was prepared according to the above general procedure. Product **4c** was isolated as a colorless liquid (14.9 mg, 38%) by silica gel chromatography using pentane/dichloromethane 7:1 solvent system as eluent. ^1^H NMR (400 MHz, CDCl_3_) δ 7.79 (d, *J* = 8.5 Hz, 2H), 7.61 (d, *J* = 8.4 Hz, 2H), 6.25 (ddq, *J* = 15.8, 8.6, 2.1 Hz, 1H), 5.65 (dq, *J* = 15.8, 6.3 Hz, 1H), 2.94–3.05 (m, 2H), 2.84–2.91 (m, 1H), 1.47–1.55 (m, 1H), 1.36–1.45 (m, 1H), 1.22–1.33 (m, 8H), 0.87 (t, *J* = 6.7 Hz, 3H). ^13^C{^1^H} NMR (101 MHz, CDCl_3_) δ 197.1, 143.1 (q, *J* = 6.4 Hz), 135.8, 132.1, 129.6, 128.6, 123.0 (q, *J* = 269.5 Hz), 119.1 (q, *J* = 33.3 Hz), 43.0, 37.2, 34.1, 31.8, 29.2, 27.1, 22.7, 14.1. ^19^F NMR (377 MHz, CDCl_3_) δ −63.92 (dt, *J* = 6.3, 1.5 Hz). HRMS (ESI-TOF) *m*/*z*: [M + Na]^+^ Calcd for C_18_H_22_BrF_3_ONa 413.0698; Found: 413.0682.

#### (R,E)-1-(4-Fluorophenyl)-3-(3,3,3-trifluoroprop-1-en-1-yl)nonan-1-one (**4d**)

This compound was prepared according to the above general procedure. Product **4d** was isolated as a colorless liquid (15.0 mg, 45%) by silica gel chromatography using a pentane/dichloromethane 5:1 solvent system as the eluent. ^1^H NMR (400 MHz, CDCl_3_) δ 7.93–7.98 (m, 2H), 7.11–7.17 (m, 2H), 6.25 (ddq, *J* = 15.8, 8.5, 2.2 Hz, 1H), 5.65 (dq, *J* = 15.8, 6.3 Hz, 1H), 2.95–3.06 (m, 2H), 2.85–2.94 (m, 1H), 1.48–1.55 (m, 1H), 1.37–1.46 (m, 1H), 1.23–1.33 (m, 8H), 0.87 (t, *J* = 6.7 Hz, 1H). ^13^C{^1^H} NMR (101 MHz, CDCl_3_) δ 196.6, 166.0 (d, *J* = 255.1 Hz), 143.2 (q, *J* = 6.4 Hz),133.6 (d, *J* = 3.1 Hz), 130.8 (d, *J* = 9.2 Hz), 123.1 (q, *J* = 269.4 Hz), 119.0 (q, *J* = 33.3 Hz), 115.9 (d, *J* = 21.8 Hz), 43.0, 37.3, 34.2, 31.8, 29.3, 27.1, 22.7, 14.2 ^19^F NMR (377 MHz, CDCl_3_) δ −63.93 (dt, *J* = 6.6, 1.5 Hz), 104.95–104.87 (m). HRMS (ESI-TOF) *m*/*z*: [M + Na]^+^ Calcd for C_18_H_22_F_4_ONa 353.1499; Found 353.1500.

#### (R,E)-1-(4-(Trifluoromethyl)phenyl)-3-(3,3,3-trifluoroprop-1-en-1-yl)nonan-1-one (**4e**)

This compound was prepared according to the above general procedure. Product **4e** was isolated as a colorless liquid (12.8 mg, 34%) by silica gel chromatography using a pentane/dichloromethane 6:1 solvent system as an eluent. ^1^H NMR (400 MHz, CDCl_3_) δ 8.03 (d, *J* = 8.1 Hz, 2H), 7.74 (d, *J* = 8.2 Hz, 2H), 6.25 (ddq, *J* = 15.8, 8.6, 2.1 Hz, 1H), 5.66 (dq, *J* = 15.8, 6.2 Hz, 1H), 3.00–3.12 (m, 2H), 2.87–2.96 (m, 1H), 1.49–1.55 (m, 1H), 1.39–1.47 (m, 1H), 1.22–1.33 (m, 8H), 0.88 (t, *J* = 6.7 Hz, 2H). ^13^C{^1^H} NMR (101 MHz, CDCl_3_) δ 197.2, 143.0 (q, *J* = 6.3 Hz), 139.7, 134.7 (q, *J* = 32.7 Hz), 128.5, 125.9 (q, *J* = 3.8 Hz), 123.7 (d, *J* = 272.8 Hz), 123.0 (d, *J* = 269.4 Hz), 119.3 (d, *J* = 33.4 Hz), 43.4, 37.2, 34.2, 31.8, 29.3, 27.1, 22.7, 14.2. ^19^F NMR (377 MHz, CDCl_3_) δ −63.98 (dt, *J* = 6.1, 1.4 Hz). HRMS (ESI-TOF) *m*/*z*: [M + Na]^+^ Calcd for C_19_H_22_F_6_ONa 403.1467; Found 403.1465.

#### (R,E)-6,6,6-Trifluoro-3-phenethyl-1-phenylhex-4-en-1-one (**4f**)

This compound was prepared according to the above general procedure except that diazoketone **2a** was added dropwise over a period of 2.5 h followed by another 0.5 h of free stirring. Product **4f** was isolated as colorless liquid (8.4 mg, 25%) by silica gel chromatography using a pentane/dichloromethane 3,5:1 solvent system as eluent. ^1^H NMR (400 MHz, CDCl_3_) δ 7.91 (d, *J* = 7.6 Hz, 2H), 7.56–7.59 (m, 1H), 7.44–7.48 (m, 2H), 7.26–7.30 (m, 2H), 7.15–7.21 (m, 3H), 6.32 (ddq, *J* = 15.8, 8.7, 2.1 Hz, 1H), 5.69 (dq, *J* = 15.8, 6.3 Hz, 1H), 3.02–3.08 (m, 2H), 2.93–3.00 (m, 1H), 2.56–2.72 (m, 2H), 1.86–1.95 (m, 1H), 1.71–1.80 (m, 1H). ^13^C{^1^H} NMR (101 MHz, CDCl_3_) δ 197.9, 142.8 (q, *J* = 6.3 Hz), 141.4, 137.0, 133.4, 128.8, 128.6, 128.4, 128.1, 126.2, 123.0 (q, *J* = 269.3 Hz), 119.6 (q, *J* = 33.2 Hz), 43.0, 36.9, 35.7, 33.4. ^19^F NMR (377 MHz, CDCl_3_) δ −63.94 (dt, *J* = 6.3, 1.5 Hz). HRMS (ESI-TOF) *m*/*z*: [M + Na]^+^ Calcd for C_20_H_19_F_3_ONa 355.1280; Found 355.1274.

#### (R,E)-1-(4-(*tert*-Butyl)phenyl)-6,6,6-trifluoro-3-phenethylhex-4-en-1-one (**4g**)

This compound was prepared according to the above general procedure, except that diazoketone **2b** was added dropwise over a period of 2.5 h, followed by another 0.5 h of free stirring. Product **4g** was isolated as a colorless liquid (9.4 mg, 24%) by silica gel chromatography using a pentane/dichloromethane 3.5:1 solvent system as eluent. ^1^H NMR (400 MHz, CDCl_3_) δ 7.85 (d, *J* = 8.3 Hz, 2H), 7.48 (d, *J* = 8.3 Hz, 2H), 7.26–7.30 (m, 2H), 7.15–7.21 (m, 3H), 6.32 (ddq, *J* = 15.8, 6.6, 2.3 Hz, 1H), 5.69 (dq, *J* = 15.8, 6.3 Hz, 1H), 3.01–3.11 (m, 2H), 2.93–3.00 (m, 1H), 2.55–2.71 (m, 2H), 1.86–1.94 (m, 1H), 1.70–1.80 (m, 1H), 1.34 (s, 9H). ^13^C{^1^H} NMR (101 MHz, CDCl_3_) δ 197.5, 157.2, 142.9 (q, *J* = 6.4 Hz), 141.4, 134.4, 128.6, 128.4, 128.1, 126.1, 125.7, 123.0 (d, *J* = 269.5 Hz), 119.4 (q, *J* = 33.4 Hz), 42.9, 36.9, 35.7, 35.2, 33.4, 31.2. ^19^F NMR (377 MHz, CDCl_3_) δ −63.91 (dt, *J* = 6.2, 1.4 Hz). HRMS (ESI-TOF) *m*/*z*: [M + Na]^+^ Calcd for C_24_H_27_F_3_ONa 411.1906; Found 411.1903.

## Data Availability

The data underlying this study are available in the published article and its Supporting Information.

## Supporting Information Available

Experimental details, general procedures, NMR spectra, SFC characterization data (PDF)
