# Supplementary material for: Stereoselective Copper-Catalyzed Cross-Coupling of α-CF3-Allylboronic Acids with Diazoketones
Source: J Org Chem. 2025 Jan 30;90(6):2542–6. doi: 10.1021/acs.joc.4c02869 (PMC11833876; doi:10.1021/acs.joc.4c02869)
Supplement: Supplementary file 1 — jo4c02869_si_001.pdf [file jo4c02869_si_001.pdf]

Supporting information

Stereoselective Copper-Catalyzed Cross-Coupling of α-CF_3-Allylboronic Acids with Diazoketones

Tautvydas Kireilis, Kálmán J. Szabó*

*Corresponding author. E-mail: kalman.j.szabo@su.se

Contents

1. General information S2

2. Enantiomeric excess analysis S3

3. NMR spectra S10

4. References S22

1. General information

Chiral SFC analysis: Unless otherwise stated, all enantiomeric excess analysis was performed using chiral SFC. Chiral SFC analysis was performed with Chiralpak IB N-3 column using MeOH/CO_2 or *i*PrOH/CO_2 as an eluent and monitored by DAD (Diode Array Detector).

Chiral HPLC analysis: Enantiomeric excess analysis was performed on products **4d** and **4e** using chiral HPLC equipped with OD-H column, and *i*PrOH/hexane or hexane was used as an eluent.

HRMS analysis: Molecular weights of the products **4a-4g** were recorded using HRMS spectrometer equipped with ESI source and time-of-flight detector.

Preparation of racemates for enantiomeric excess analysis: The enantiomers of allylboronic acid **1** (S) and (R) were prepared according to the published procedure,[1] and used in the following general procedure A to give corresponding products **4-**(*R*) and **4**-(*S*). Racemic mixtures were prepared by combining equimolar amounts of product **4**-(*R*) and **4**-(*S*). The minor deviations from 50/50 ratio of the S/R enantiomers may be due to weighing errors.

2. Enantiomeric excess analysis

(R,E)-1-phenyl-3-(3,3,3-trifluoroprop-1-en-1-yl)nonan-1-one (4a):

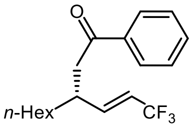

Determination of *ee*: Chiral SFC (Daicel CHIRALPAK IB N-3 column, CO_2/MeOH = 97.5/2.5, flow rate = 1.0 mL/min, l = 230 nm) tR = 6.00 min (minor), 6.52 min (major); *ee* = 95%.

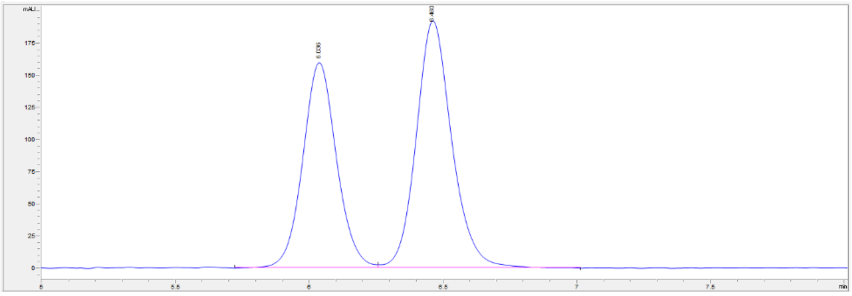

Signal 2: DAD1 B, Sig=230,4 Ref=off

Peak #	RetTime [min]	Type	Width [min]	Area [mAU*s]	Height [mAU]	Area %
1	6.036	BV	0.1346	1411.86121	160.59169	44.0421
2	6.460	VB	0.1403	1793.84485	193.29440	55.9579
Totals :				3205.70605	353.88609	

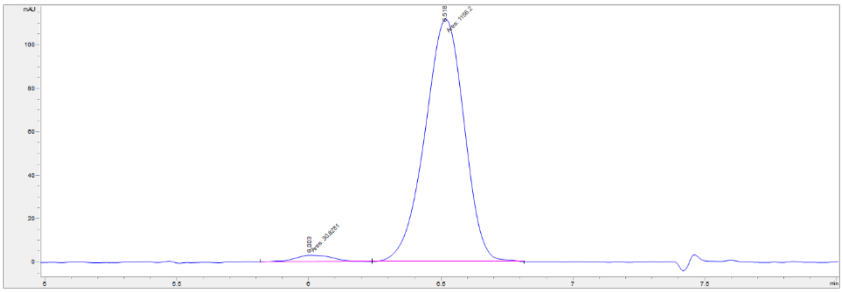

Signal 2: DAD1 B, Sig=230,4 Ref=off

Peak #	RetTime [min]	Type	Width [min]	Area [mAU*s]	Height [mAU]	Area %
1	6.003	MM	0.1700	30.82815	3.02280	2.5971
2	6.518	MM	0.1721	1156.20068	111.94198	97.4029
Totals :				1187.02883	114.96478	

(R,E)-1-(4-(tert-butyl)phenyl)-3-(3,3,3-trifluoroprop-1-en-1-yl)nonan-1-one (4b):

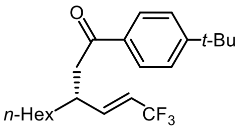

Determination of *ee*: Chiral SFC (Daicel CHIRALPAK IB N-3 column, CO_2/MeOH = 98/2, flow rate = 1.0 mL/min, l = 254 nm) tR = 8.09 min (minor), 9.53 min (major); *ee* = 95%.

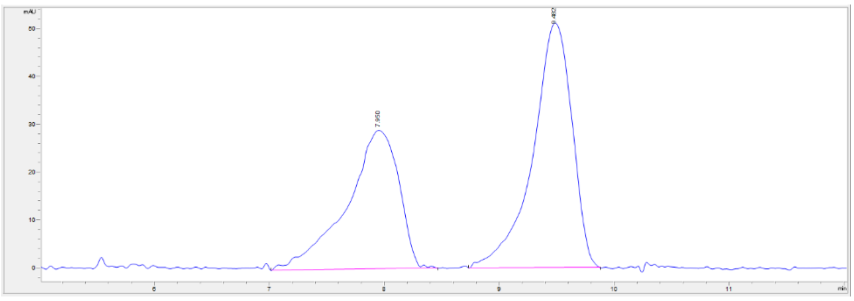

Signal 1: DAD1 A, Sig=254,4 Ref=off

Peak #	RetTime [min]	Type	Width [min]	Area [mAU*s]	Height [mAU]	Area %
1	7.950	BV R	0.4210	920.74310	29.10914	43.0039
2	9.482	BB	0.3433	1220.32446	51.33771	56.9961
Totals :				2141.06757	80.44685	

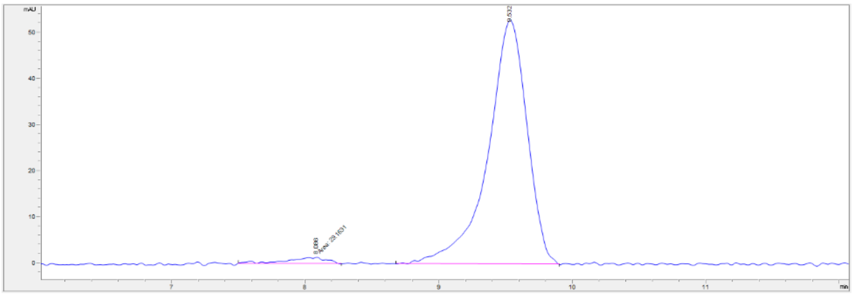

Signal 1: DAD1 A, Sig=254,4 Ref=off

Peak #	RetTime [min]	Type	Width [min]	Area [mAU*s]	Height [mAU]	Area %
1	8.086	MM	0.3384	29.16312	1.43640	2.6225
2	9.532	VB R	0.3001	1082.88745	53.07134	97.3775
Totals :				1112.05057	54.50774	

(R,E)-1-(4-bromophenyl)-3-(3,3,3-trifluoroprop-1-en-1-yl)nonan-1-one (4c):

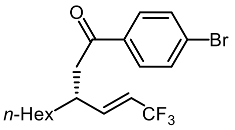

Determination of *ee*: Chiral SFC (Daicel CHIRALPAK IB N-3 column, CO_2/*i*PrOH = 98/2, flow rate = 1 mL/min, l = 254 nm) tR = 9.80 min (minor), 10.51 min (major); *ee* = 94%.

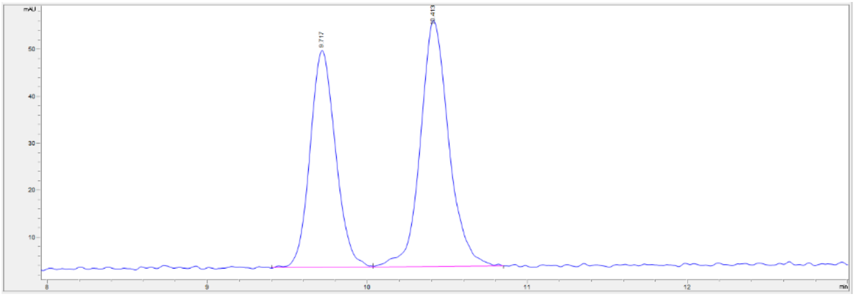

Signal 1: DAD1 A, Sig=254,4 Ref=off

Peak #	RetTime [min]	Type	Width [min]	Area [mAU*s]	Height [mAU]	Area %
1	9.717	VV R	0.1739	514.57825	46.20005	44.2195
2	10.413	VV R	0.1925	649.11353	52.39146	55.7805
Totals :				1163.69177	98.59150	

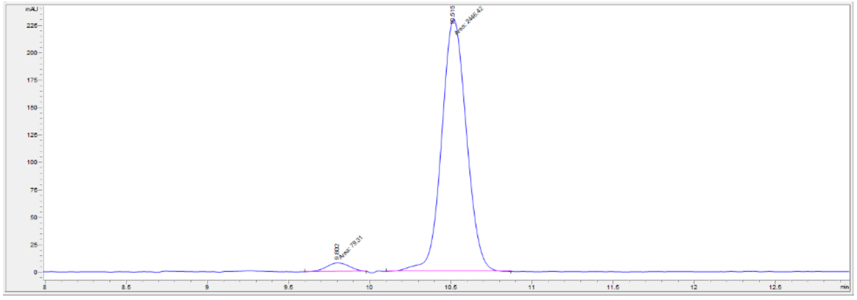

Signal 1: DAD1 A, Sig=254,4 Ref=off

Peak #	RetTime [min]	Type	Width [min]	Area [mAU*s]	Height [mAU]	Area %
1	9.802	MM	0.1681	79.31000	7.86155	3.1401
2	10.515	MM	0.1773	2446.41992	229.96294	96.8599
Totals :				2525.72992	237.82449	

(R,E)-1-(4-bromophenyl)-3-(3,3,3-trifluoroprop-1-en-1-yl)nonan-1-one (4d):

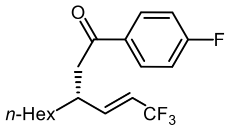

Determination of *ee*: Chiral HPLC (Daicel CHIRALCEL OD-H column, *n*-hexane/*i*PrOH = 99.9/0.1, flow rate = 0.5 mL/min, l = 250 nm) tR =19.50 min (minor), 20.61 min (major); *ee* = 95%.

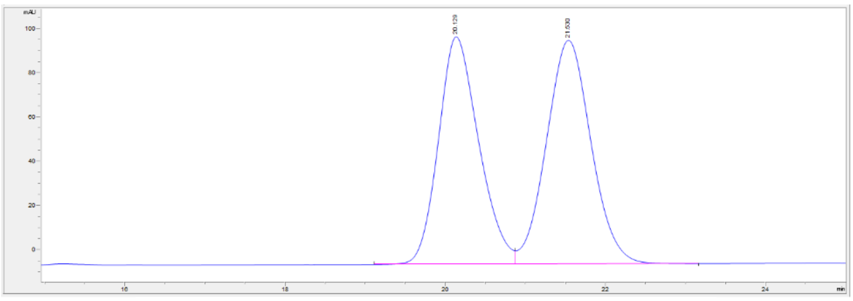

Signal 1: MWD1 A, Sig=250,4 Ref=off

Peak #	RetTime [min]	Type	Width [min]	Area [mAU*s]	Height [mAU]	Area %
1	20.129	BV	0.5264	3589.68896	102.55336	47.9265
2	21.530	VB	0.5992	3900.29272	100.84692	52.0735
Totals :				7489.98169	203.40028	

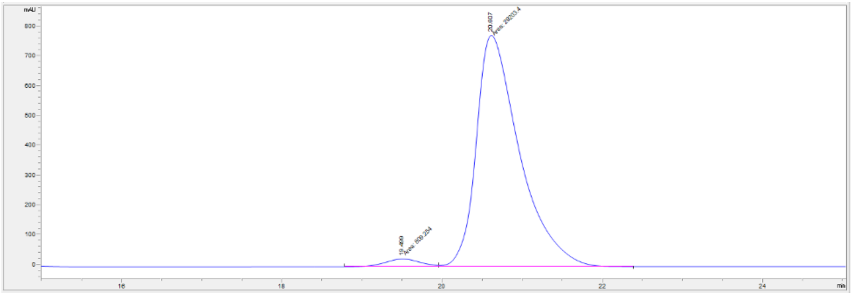

Signal 1: MWD1 A, Sig=250,4 Ref=off

Peak #	RetTime [min]	Type	Width [min]	Area [mAU*s]	Height [mAU]	Area %
1	19.499	MM	0.5088	809.25372	26.51071	2.6964
2	20.607	MM	0.6253	2.92034e4	778.33405	97.3036
Totals :				3.00127e4	804.84476	

(R,E)-1-(4-((difluoro-l3-methyl)-l2-fluoraneyl)phenyl)-3-(3,3,3-trifluoroprop-1-en-1-yl)nonan-1-one (4e):

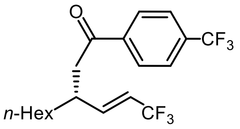

Determination of *ee*: Chiral HPLC (Daicel CHIRALCEL OD-H column, hexane as eluent, flow rate = 1.0 mL/min, l = 230 nm) tR = 14.18 min (minor), 14.76 min (major); *ee* = 94%.

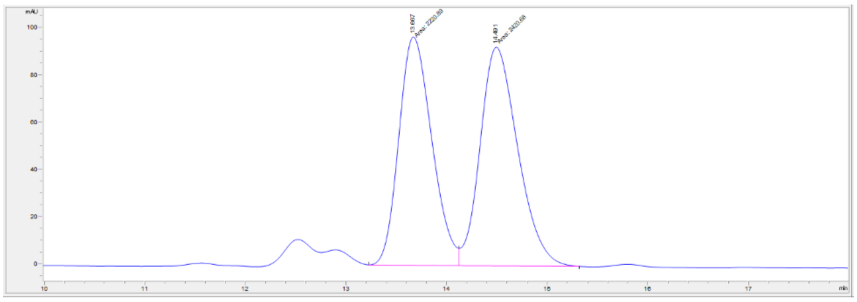

Signal 4: MWD1 D, Sig=230,4 Ref=off

Peak #	RetTime [min]	Type	Width [min]	Area [mAU*s]	Height [mAU]	Area %
1	13.667	MM	0.3822	2220.88672	96.85094	47.8480
2	14.491	MM	0.4352	2420.65527	92.69698	52.1520
Totals :				4641.54199	189.54792	

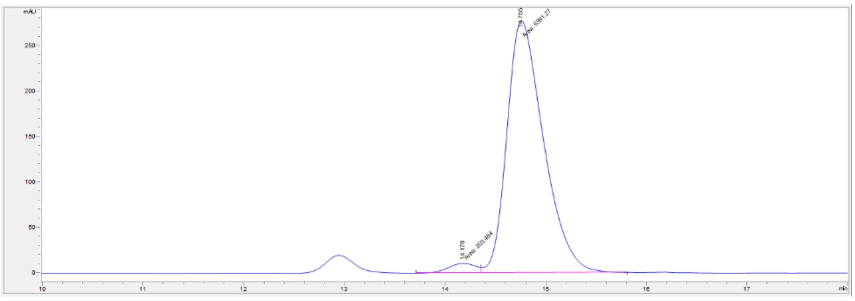

Signal 4: MWD1 D, Sig=230,4 Ref=off

Peak #	RetTime [min]	Type	Width [min]	Area [mAU*s]	Height [mAU]	Area %
1	14.178	MM	0.3195	203.46429	10.61352	2.8280
2	14.755	MM	0.4212	6991.26611	276.66779	97.1720
Totals :				7194.73041	287.28131	

(R,E)-6,6,6-trifluoro-3-phenethyl-1-phenylhex-4-en-1-one (4f):

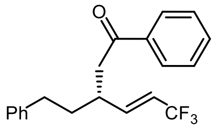

Determination of *ee*: Chiral SFC (Daicel CHIRALPAK IB N-3 column, CO_2/MeOH = 97/3, flow rate = 1 mL/min, l = 210 nm) tR: 17.09 min (minor), 18.18 min (major); *ee* = 97%.

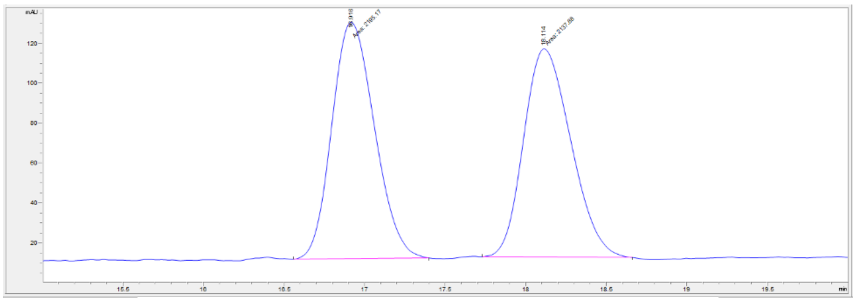

Signal 3: DAD1 C, Sig=210,4 Ref=off

Peak #	RetTime [min]	Type	Width [min]	Area [mAU*s]	Height [mAU]	Area %
1	16.916	MM	0.3082	2195.17310	118.72672	50.6611
2	18.114	MM	0.3407	2137.88110	104.58779	49.3389
Totals :				4333.05420	223.31451	

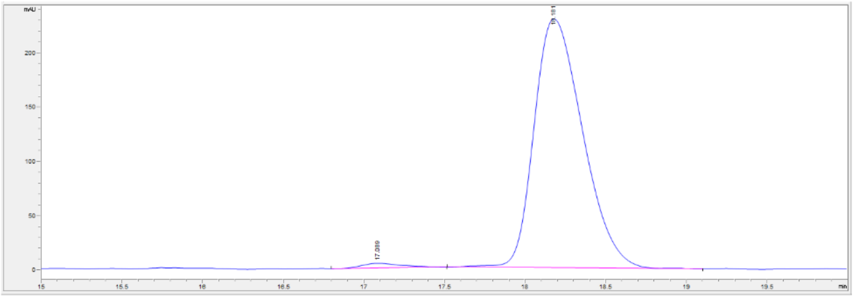

Signal 3: DAD1 C, Sig=210,4 Ref=off

Peak #	RetTime [min]	Type	Width [min]	Area [mAU*s]	Height [mAU]	Area %
1	17.089	BB	0.2489	79.84510	4.45847	1.6278
2	18.181	BV R	0.3334	4825.24463	229.44276	98.3722
Totals :				4905.08973	233.90124	

(R,E)-1-(4-(tert-butyl)phenyl)-6,6,6-trifluoro-3-phenethylhex-4-en-1-one (4g):

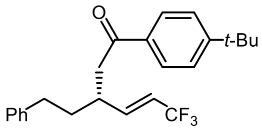

Determination of *ee*: Chiral SFC (Daicel CHIRALPAK IB N-3 column, CO_2/MeOH = 97/3, flow rate = 1 mL/min, l = 254 nm) tR = 13.02 min (major), 13.70 min (minor); *ee* = 91%.

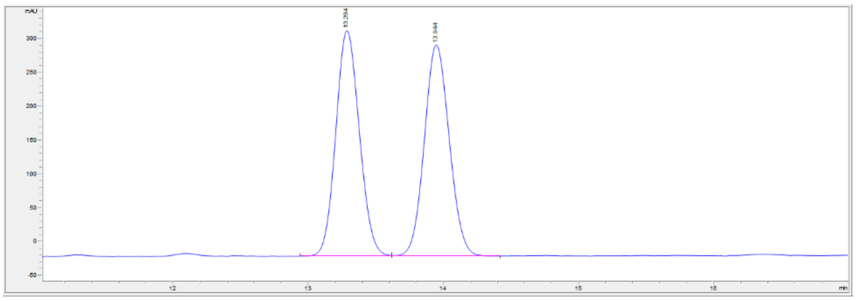

Signal 1: DAD1 A, Sig=254,4 Ref=off

Peak #	RetTime [min]	Type	Width [min]	Area [mAU*s]	Height [mAU]	Area %
1	13.284	BV	0.1891	4018.63013	332.25403	50.2195
2	13.944	VV R	0.1994	3983.49634	311.13745	49.7805
Totals :				8002.12646	643.39148	

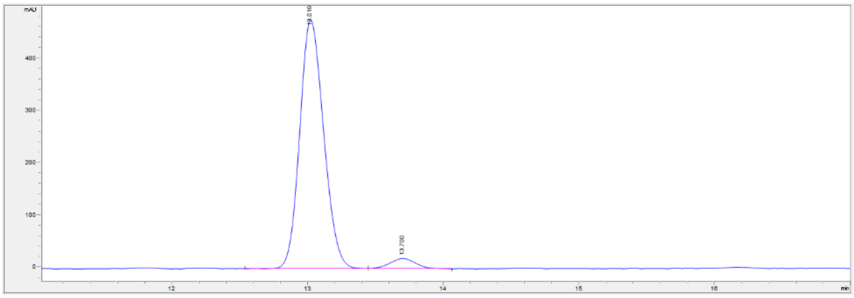

Signal 1: DAD1 A, Sig=254,4 Ref=off

Peak #	RetTime [min]	Type	Width [min]	Area [mAU*s]	Height [mAU]	Area %
1	13.019	VV R	0.1891	5853.87451	477.10501	95.6127
2	13.700	VB	0.1813	268.61343	19.91946	4.3873
Totals :				6122.48795	497.02448	

3. NMR spectra

^{1}H NMR ($CDCl_3$, 400 MHz) of compound **4a**

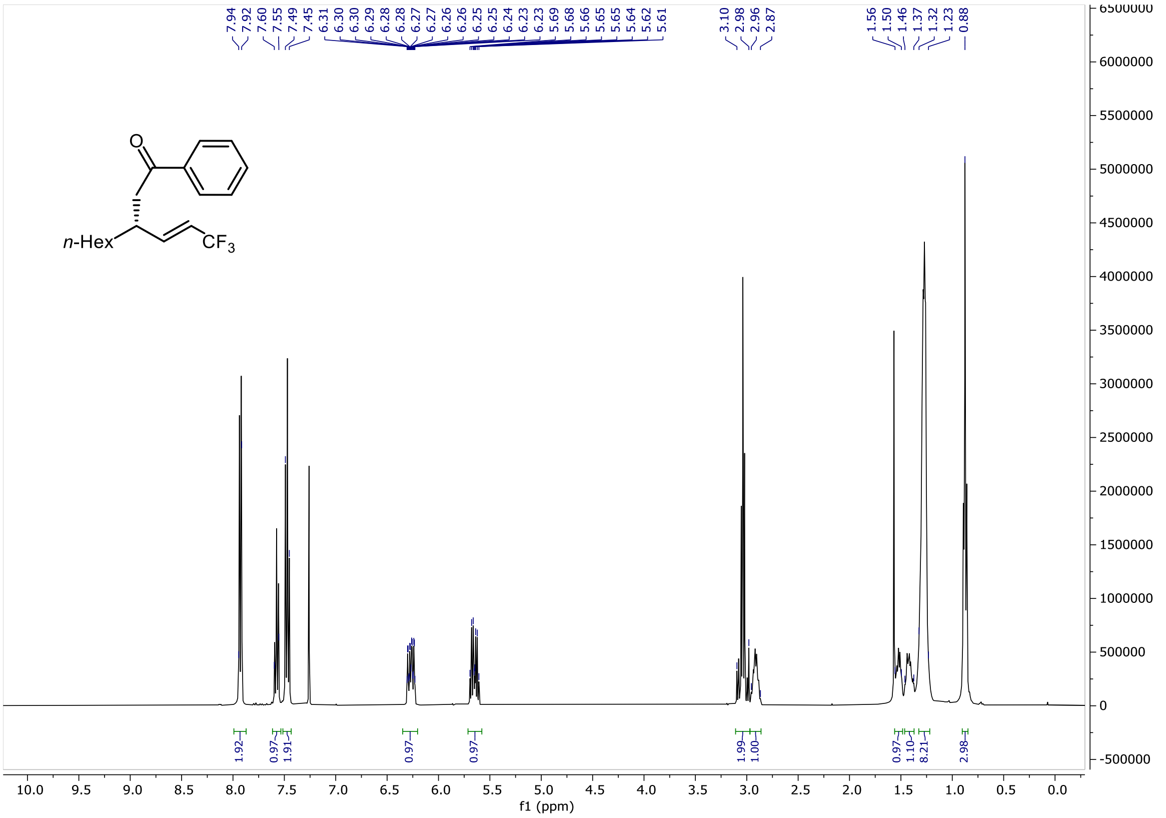

^{13}C NMR (101 MHz, $CDCl_3$) of compound **4a**

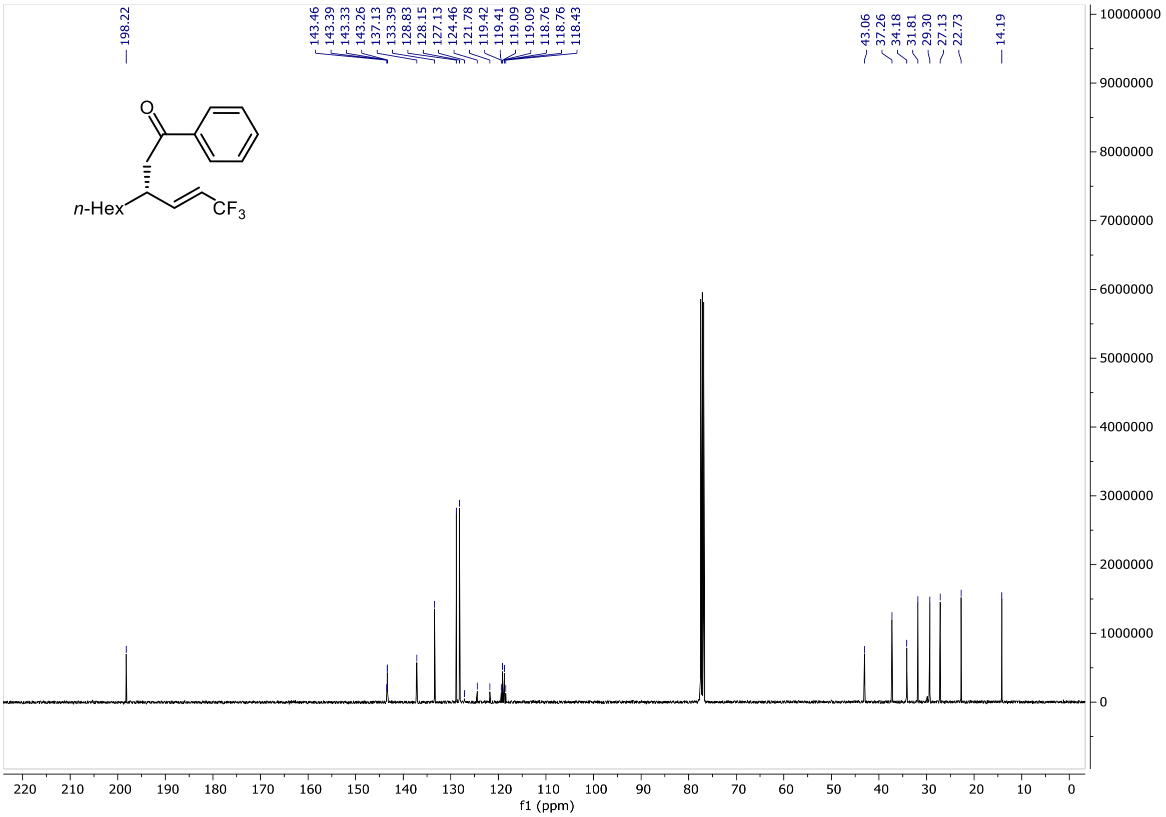

^{19}F NMR (377 MHz, $CDCl_3$) of compound **4a**

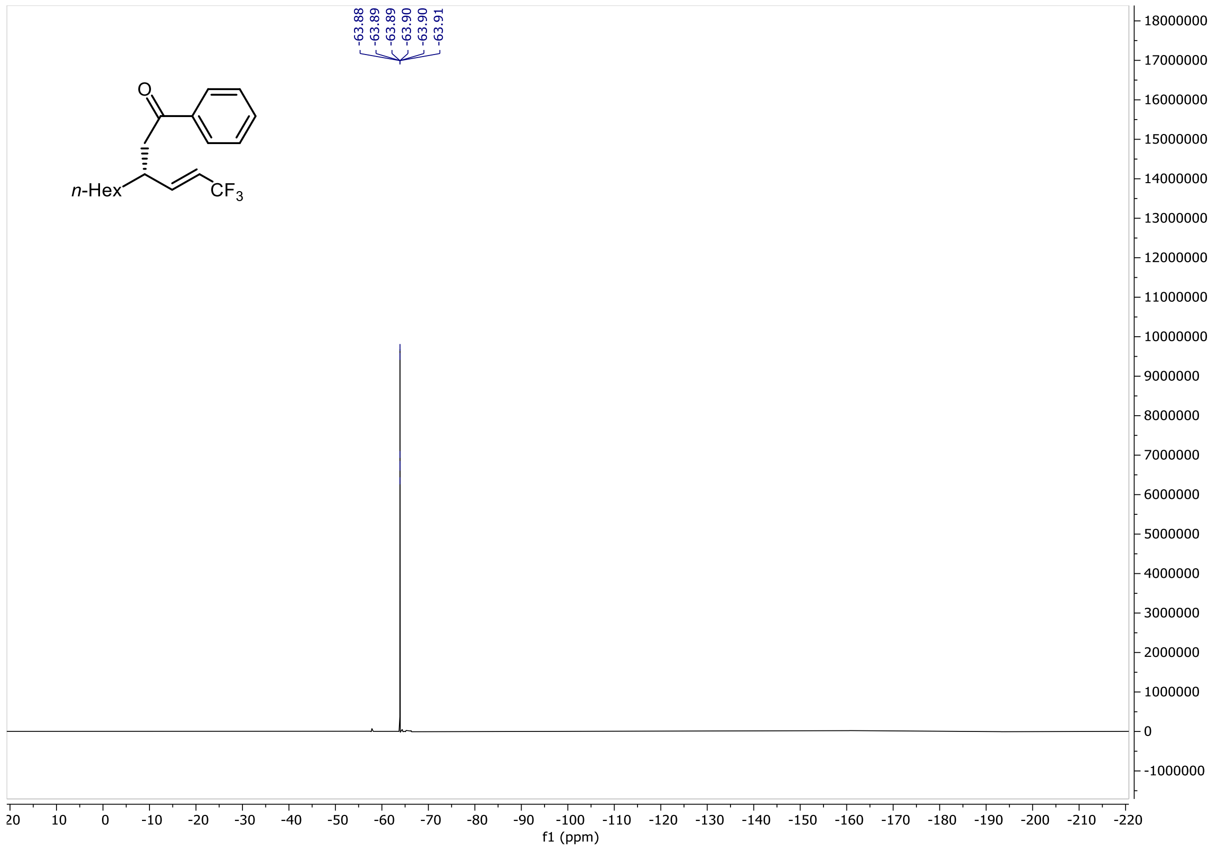

^{1}H NMR ($CDCl_3$, 400 MHz) of compound **4b**

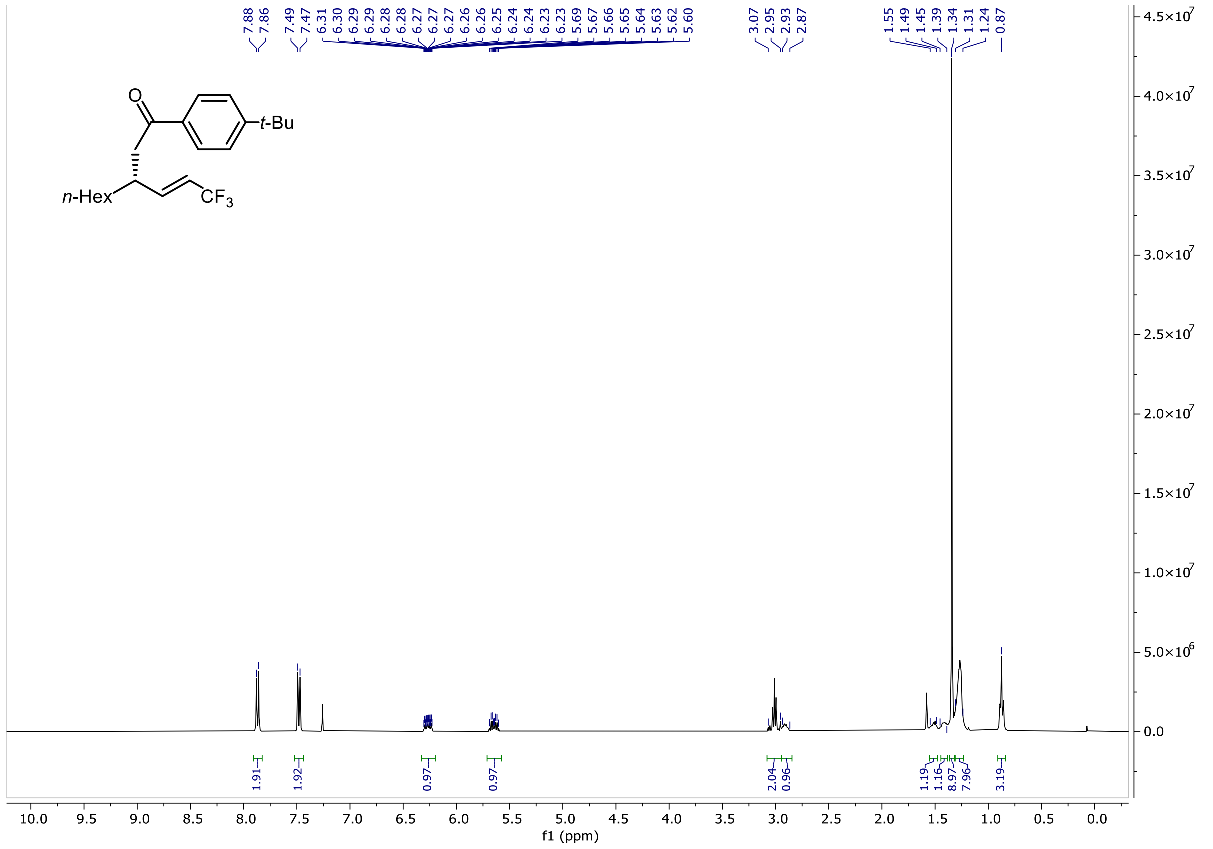

^{13}C NMR (101 MHz, $CDCl_3$) of compound **4b**

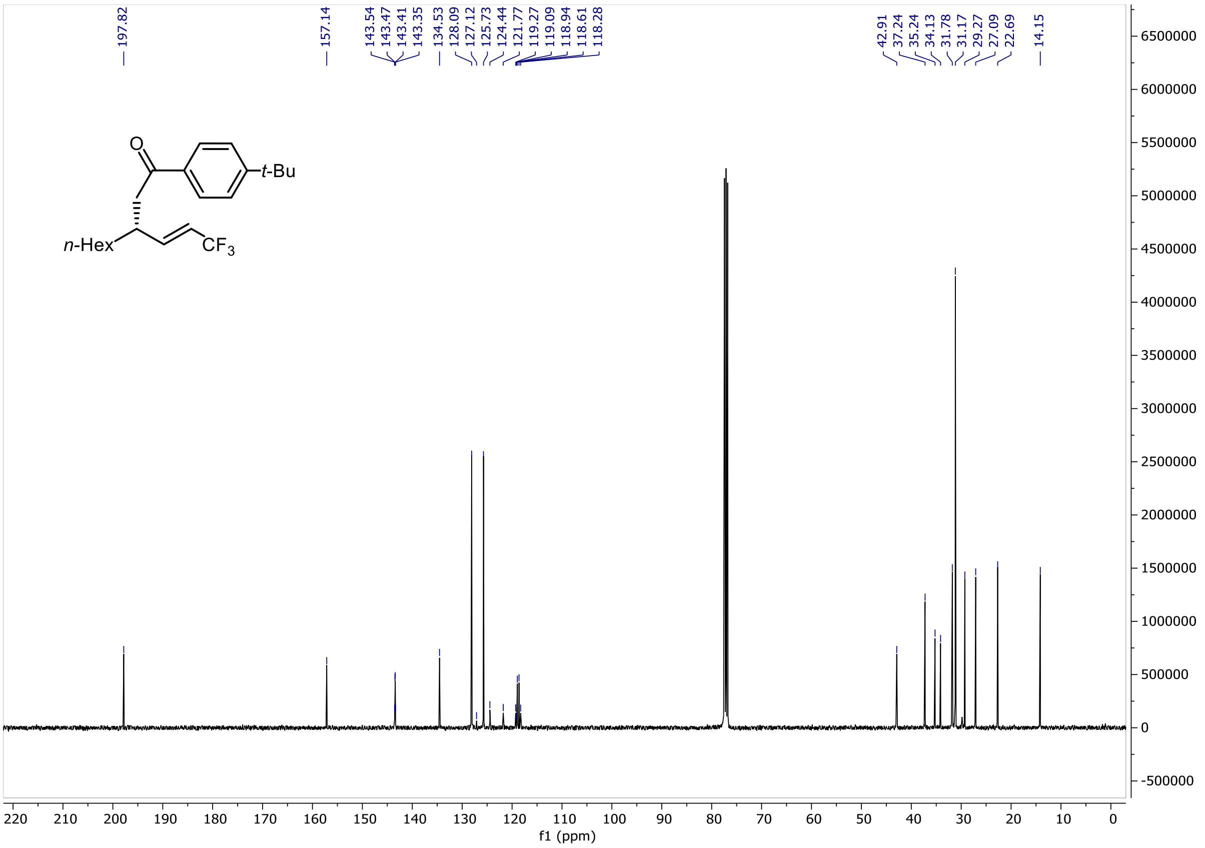

^{19}F NMR (377 MHz, $CDCl_3$) of compound **4b**

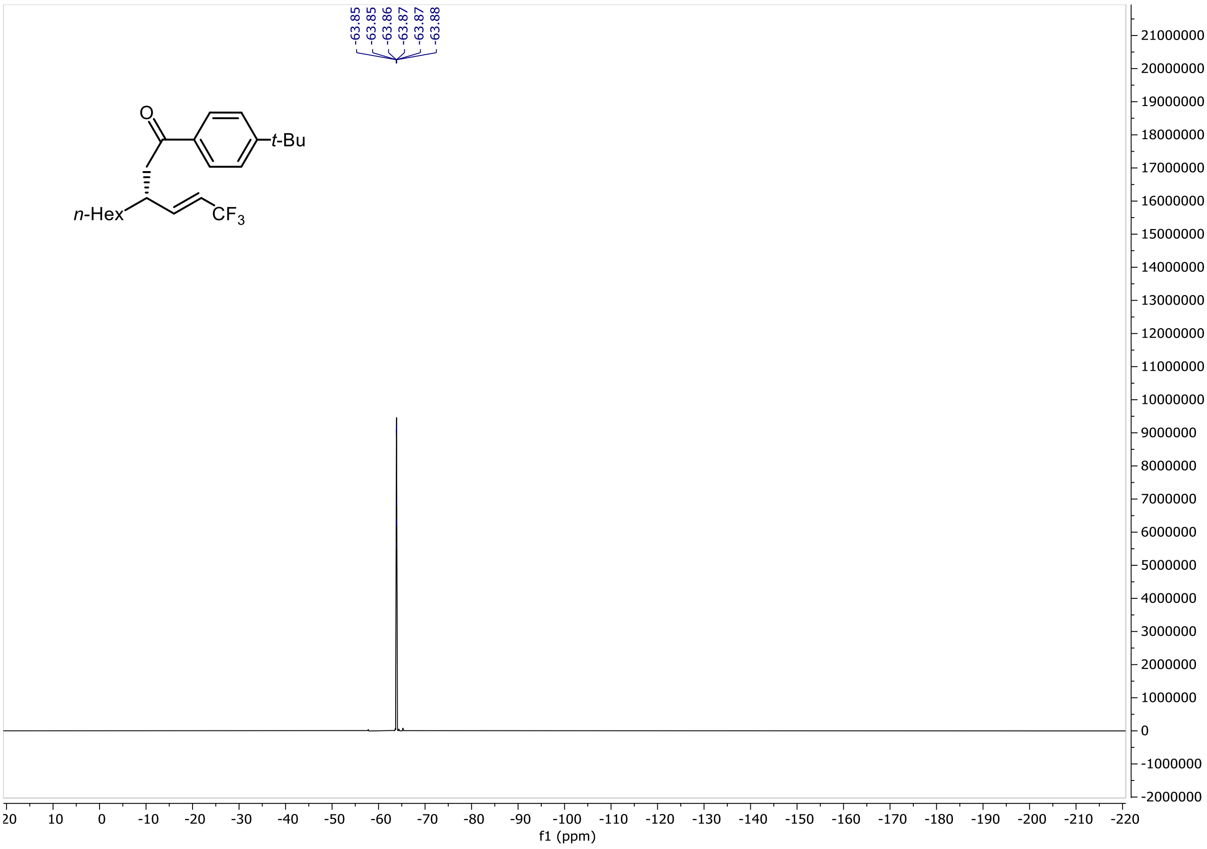

^{1}H NMR ($CDCl_3$, 400 MHz) of compound **4c**

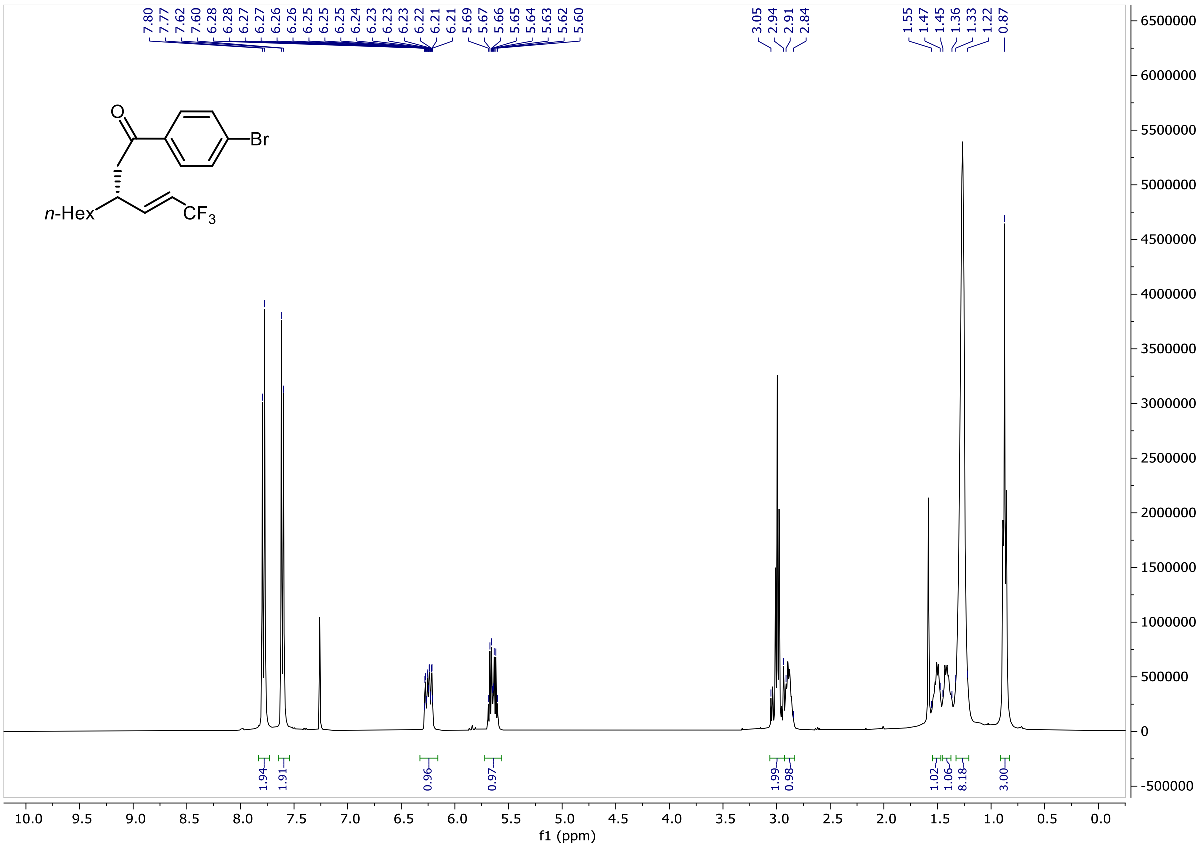

^{13}C NMR (101 MHz, $CDCl_3$) of compound **4c**

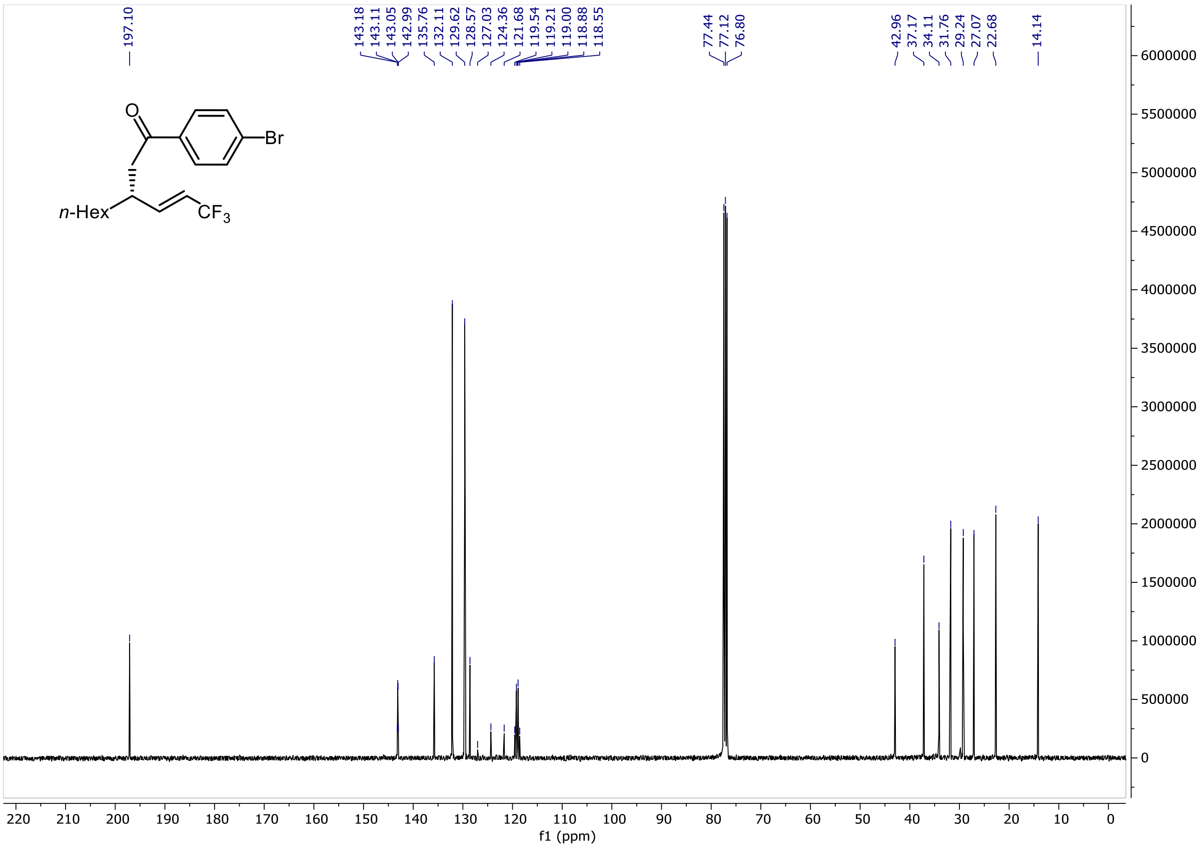

^{19}F NMR (377 MHz, $CDCl_3$) of compound **4c**

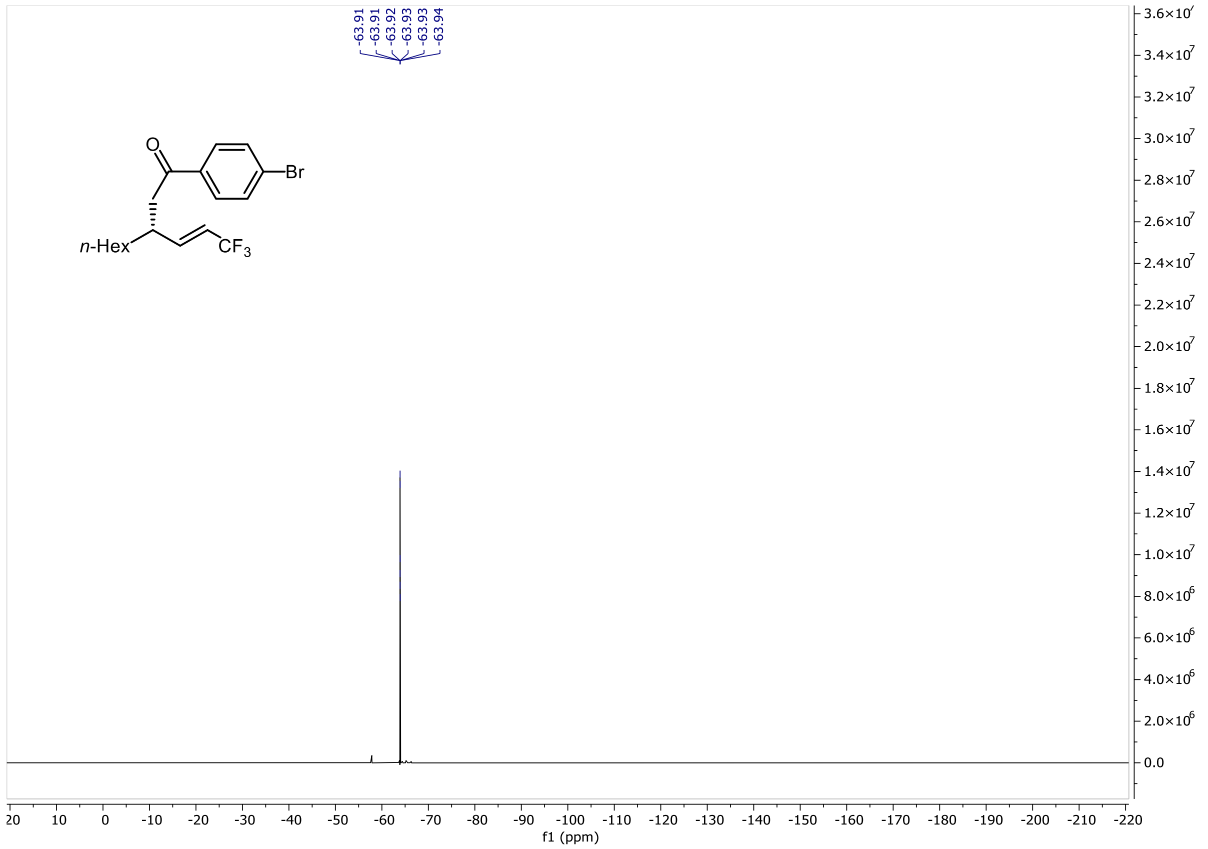

^{1}H NMR ($CDCl_3$, 400 MHz) of compound **4d**

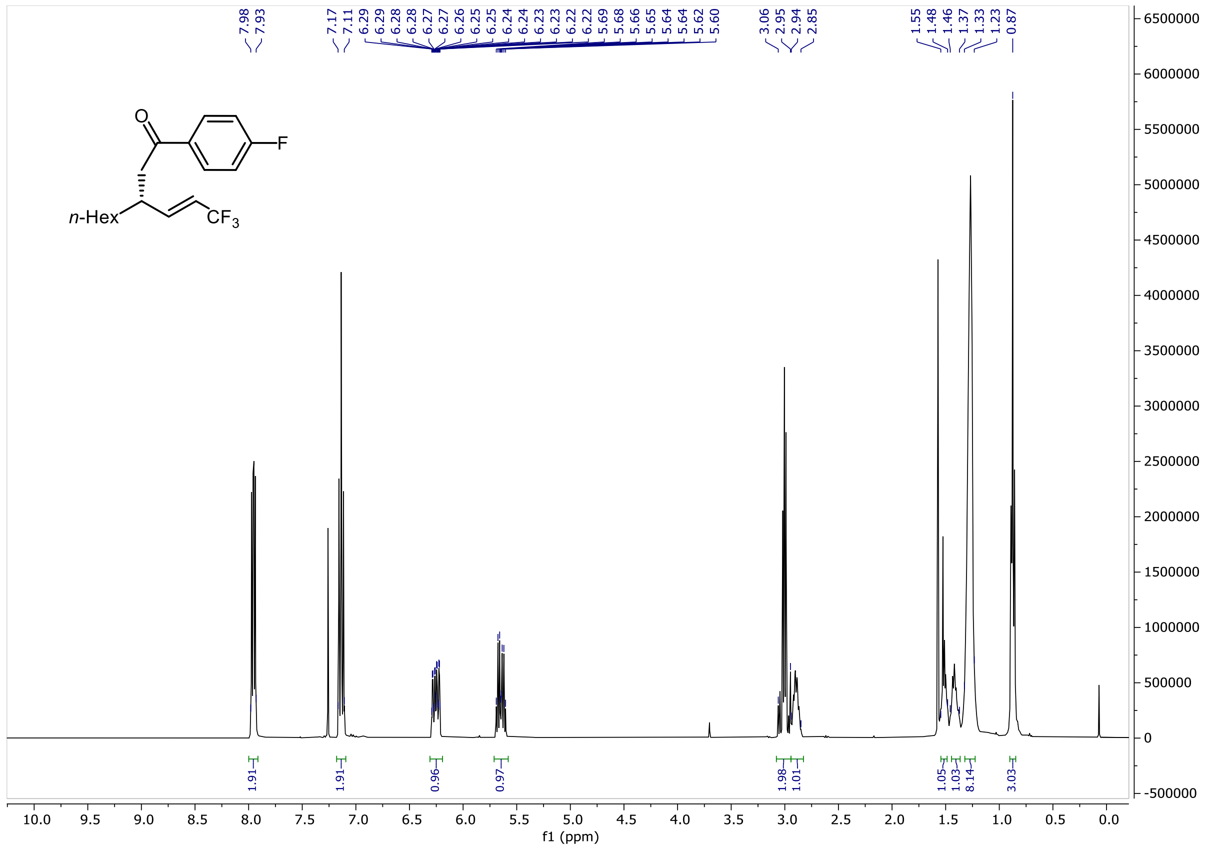

^{13}C NMR (101 MHz, $CDCl_3$) of compound **4d**

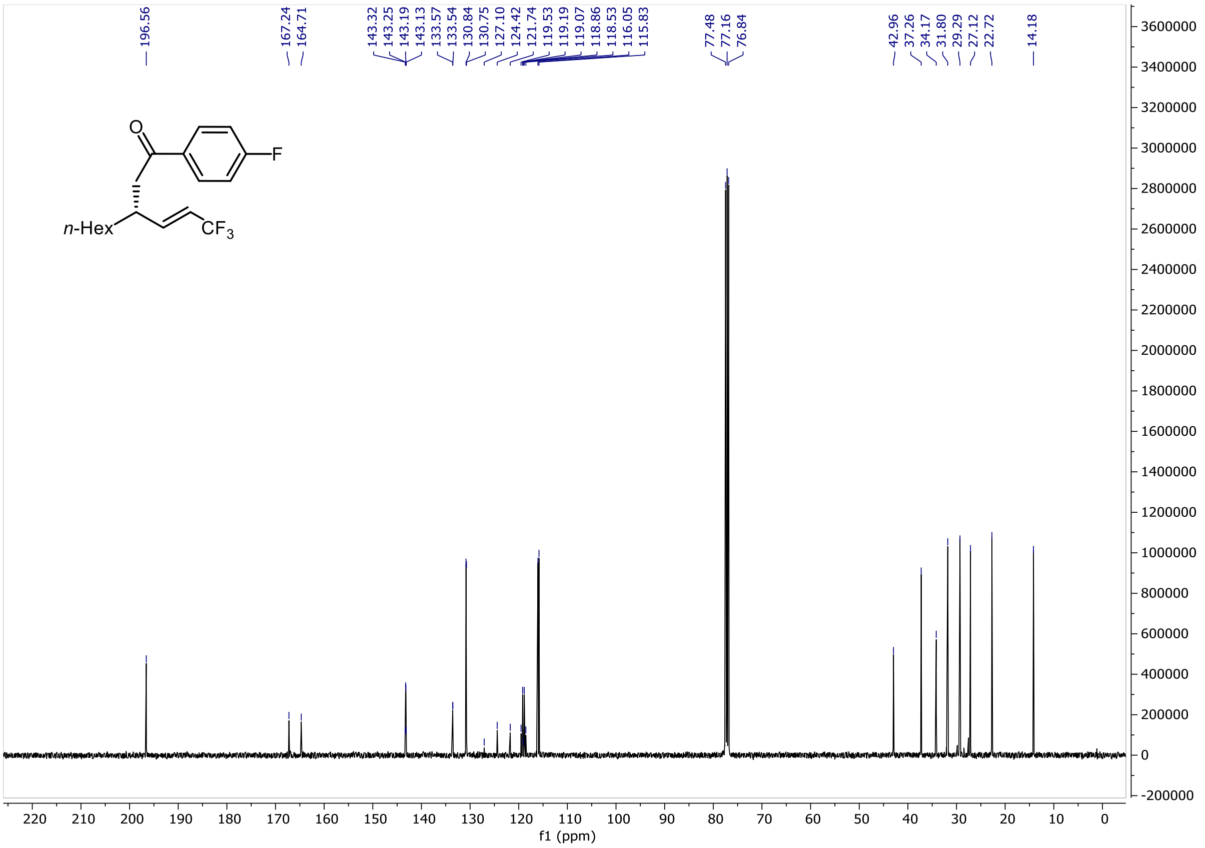

^{19}F NMR (377 MHz, $CDCl_3$) of compound **4d**

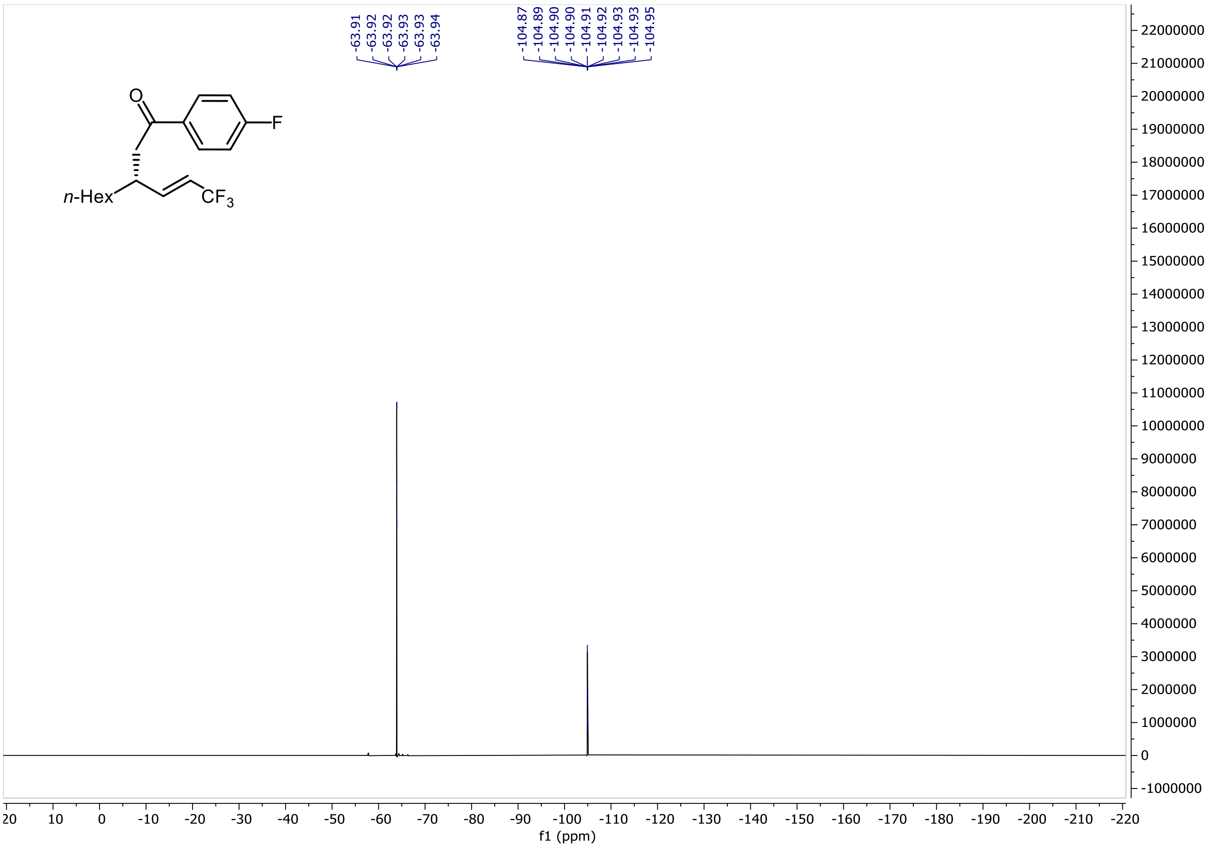

^{1}H NMR ($CDCl_3$, 400 MHz) of compound **4e**

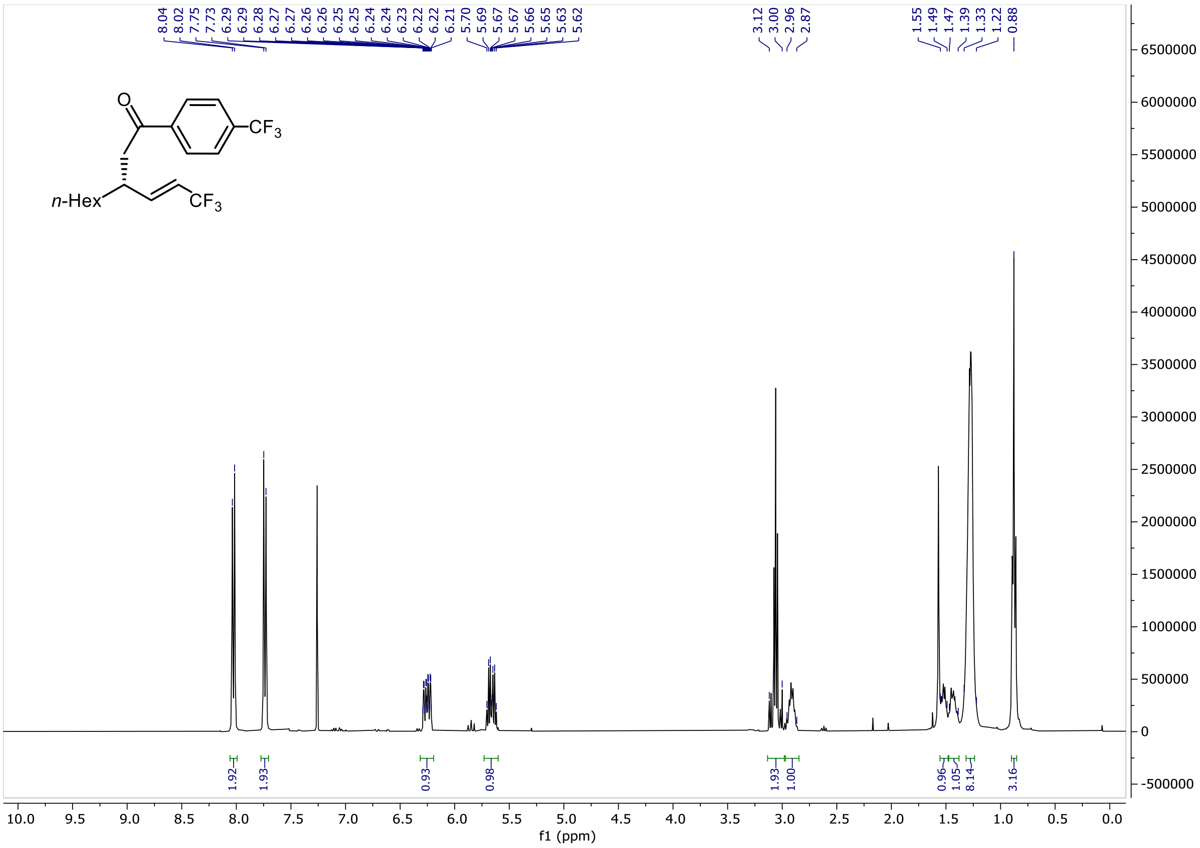

^{13}C NMR (101 MHz, $CDCl_3$) of compound **4e**

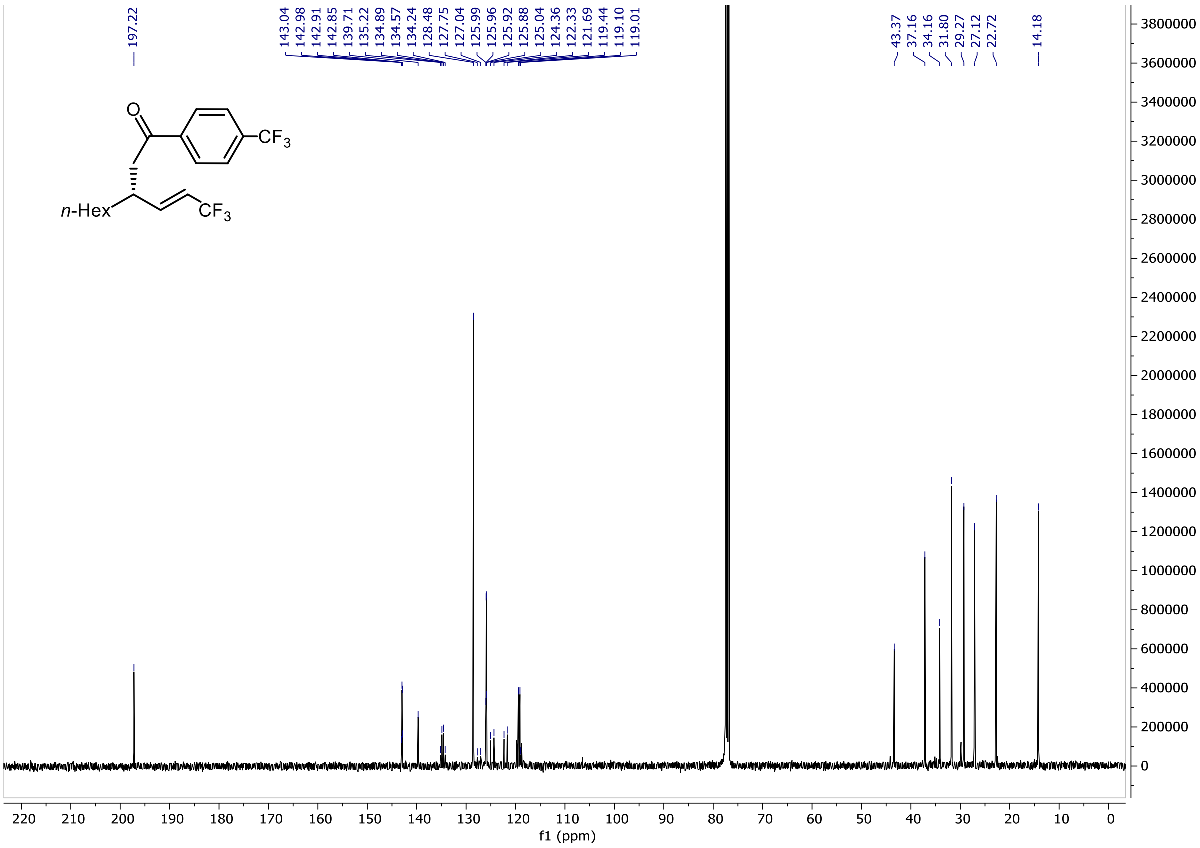

^{19}F NMR (377 MHz, $CDCl_3$) of compound **4e**

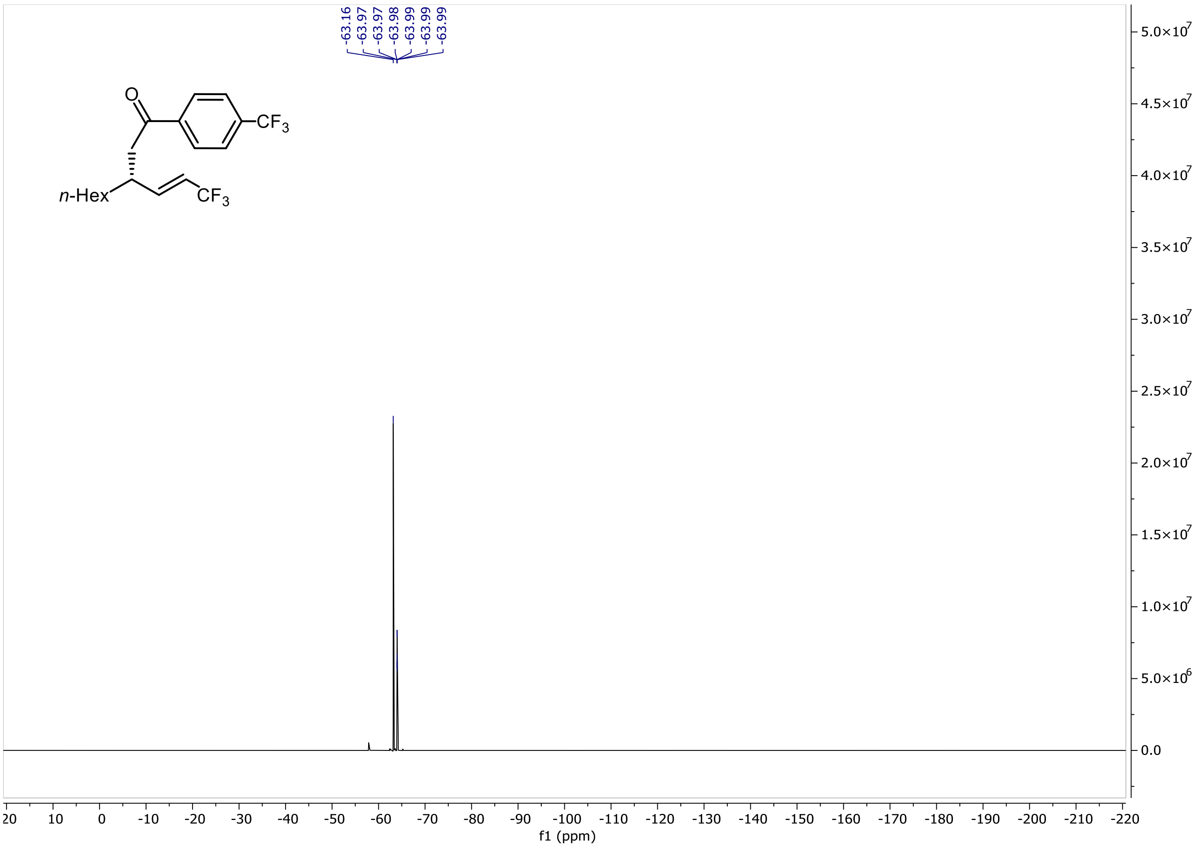

^{1}H NMR ($CDCl_3$, 400 MHz) of compound **4f**

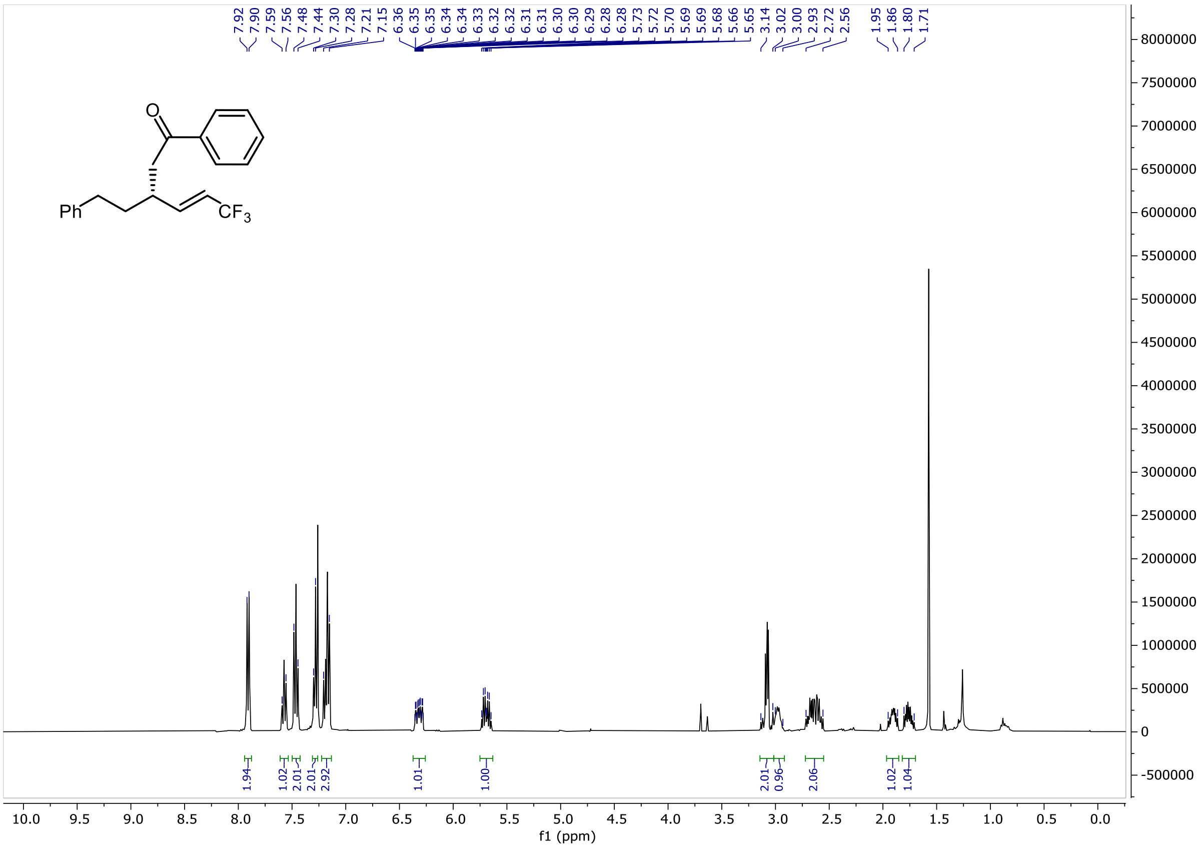

^{13}C NMR (101 MHz, $CDCl_3$) of compound **4f**

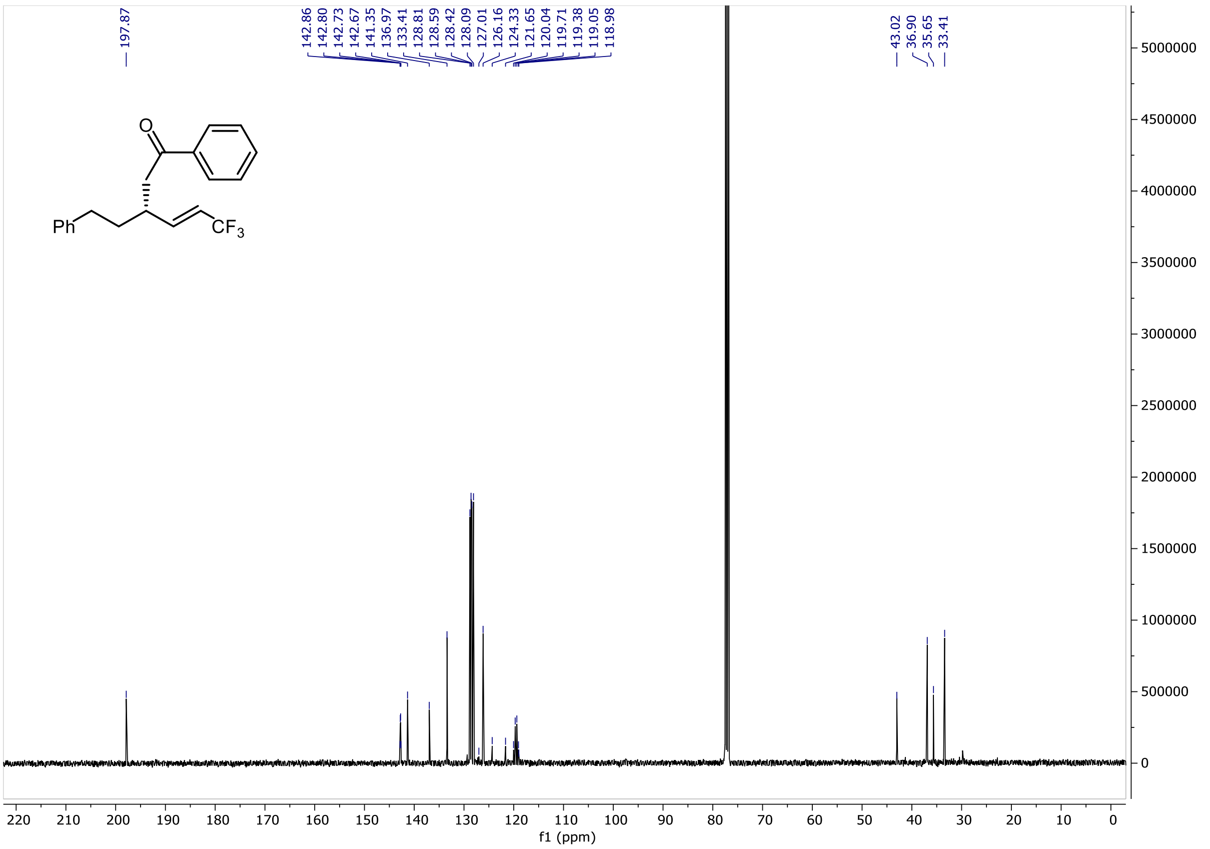

^{19}F NMR (377 MHz, $CDCl_3$) of compound **4f**

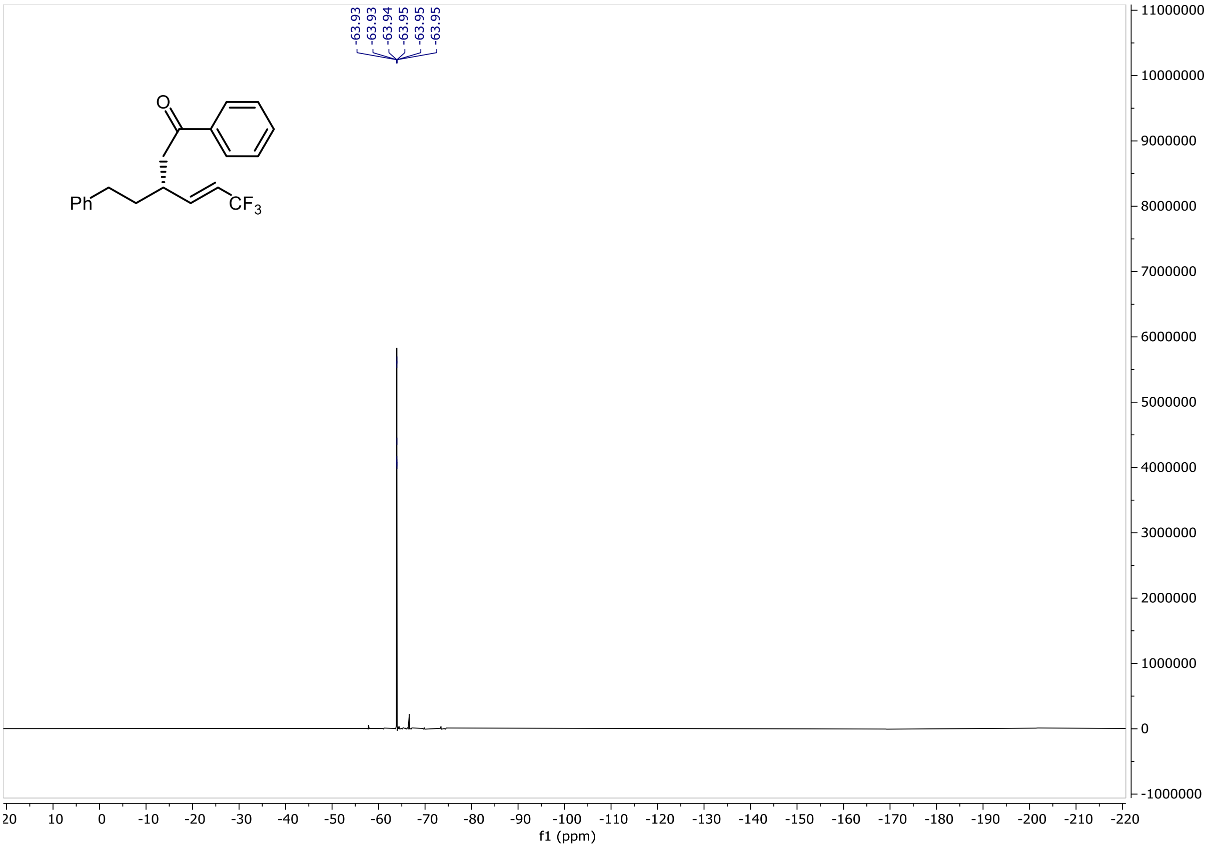

^{1}H NMR ($CDCl_3$, 400 MHz) of compound **4g**

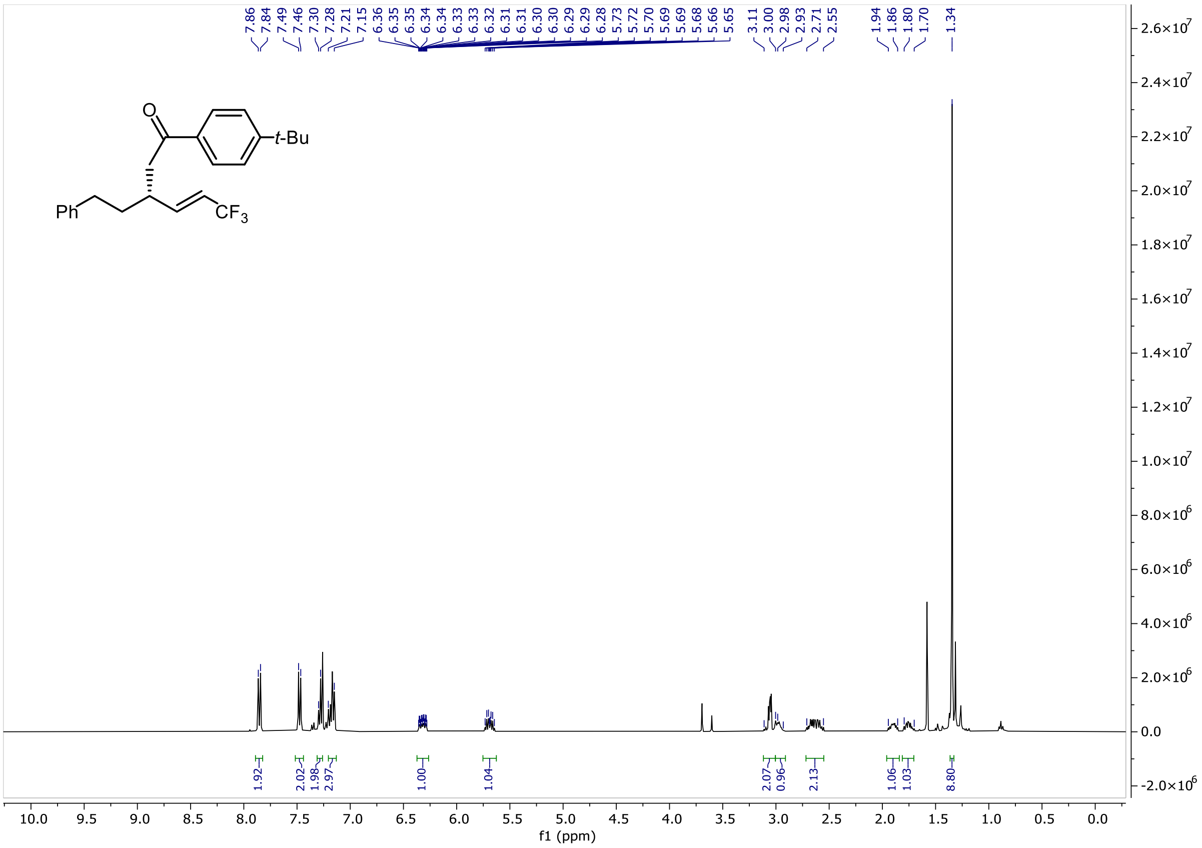

^{13}C NMR (101 MHz, $CDCl_3$) of compound **4g**

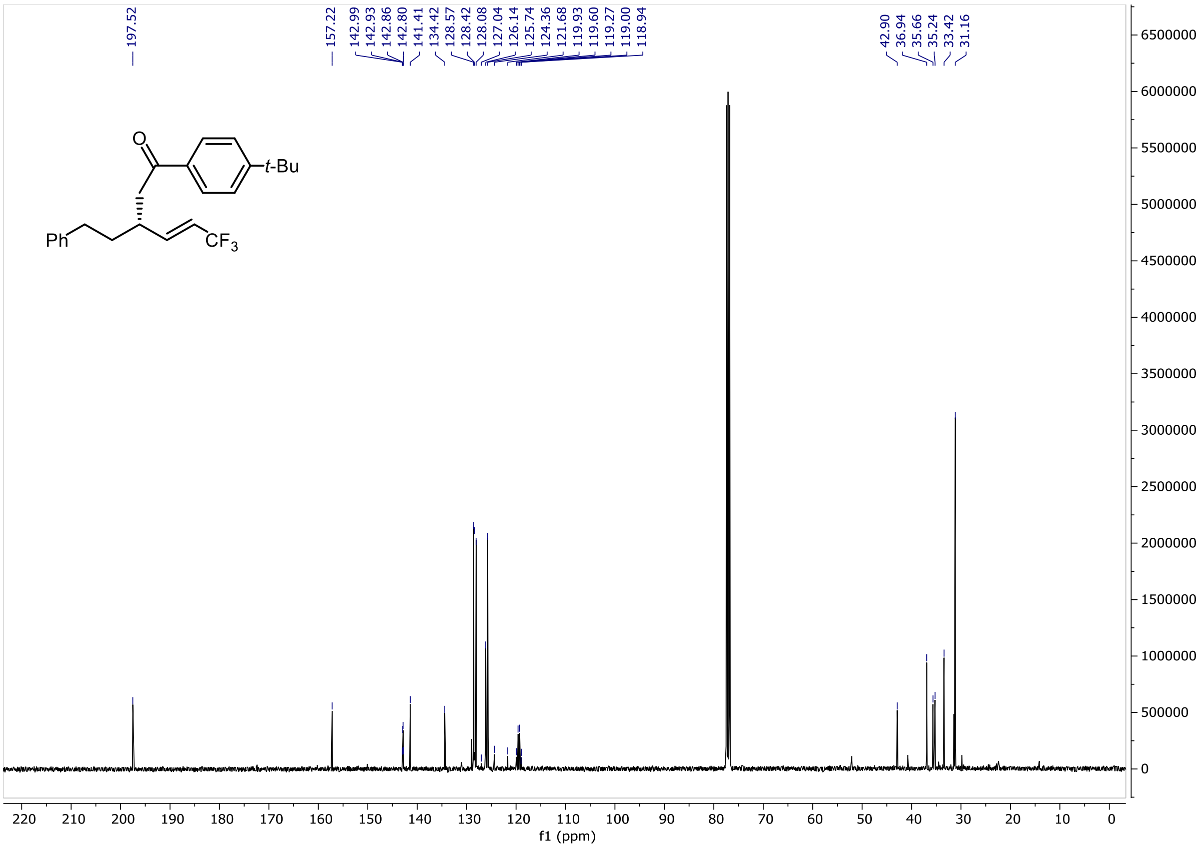

^{19}F NMR (377 MHz, $CDCl_3$) of compound **4g**

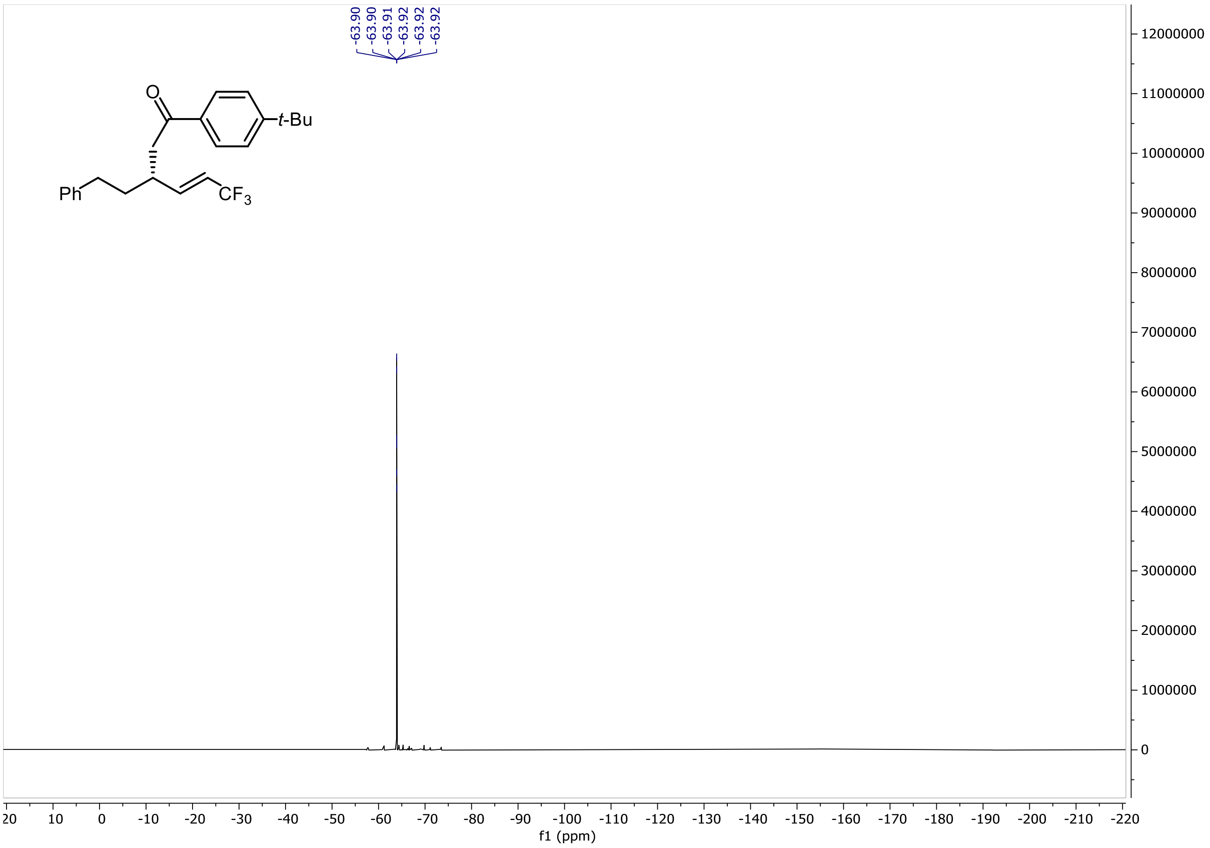

4. References

[1] S. J. T. Jonker, R. Jayarajan, T. Kireilis, M. Deliaval, L. Eriksson, K. J. Szabó, *J. Am. Chem. Soc.* **2020**, *142*, 21254-21259.
